# Causal Mediation Analysis: A Summary‐Data Mendelian Randomization Approach

**DOI:** 10.1002/sim.10317

**Published:** 2025-02-05

**Authors:** Shu‐Chin Lin, Sheng‐Hsuan Lin, Tian Ge, Chia‐Yen Chen, Yen‐Feng Lin

**Affiliations:** ^1^ Center for Neuropsychiatric Research National Health Research Institutes Miaoli Taiwan; ^2^ Institute of Statistics and Data Science National Taiwan University Taipei Taiwan; ^3^ Institute of Statistics National Yang Ming Chiao Tung University Hsinchu Taiwan; ^4^ Psychiatric and Neurodevelopmental Genetics Unit, Center for Genomic Medicine Massachusetts General Hospital Boston Massachusetts USA; ^5^ Stanley Center for Psychiatric Research Broad Institute of MIT and Harvard Cambridge Massachusetts USA; ^6^ Department of Psychiatry Massachusetts General Hospital, Harvard Medical School Boston Massachusetts USA; ^7^ Translational Medicine Biogen Cambridge Massachusetts USA; ^8^ Department of Public Health & Medical Humanities, School of Medicine National Yang Ming Chiao Tung University Taipei Taiwan; ^9^ Institute of Behavioral Medicine, College of Medicine National Cheng Kung University Tainan Taiwan

**Keywords:** causal inference, indirect effect, mediation analysis, mediation proportion, summary‐data Mendelian randomization

## Abstract

Summary‐data Mendelian randomization (MR), a widely used approach in causal inference, has recently attracted attention for improving causal mediation analysis. Two existing methods corresponding to the difference method and product method of linear mediation analysis have been developed to perform MR‐based mediation analysis using the inverse‐variance weighted method (MR‐IVW). Despite these developments, there is still a need for more rigorous, efficient, and precise MR‐based mediation methodologies. In this study, we develop summary‐data MR‐based frameworks for causal mediation analysis. We improve the accuracy, statistical efficiency and robustness of the existing MR‐based mediation analysis by implementing novel variance estimators for the mediation effects, deriving rigorous procedures for statistical inference, and accounting for widespread pleiotropic effects. Specifically, we propose Diff‐IVW and Prod‐IVW to improve upon the existing methods and provide the pleiotropy‐robust methods (Diff‐Egger, Diff‐Median, Prod‐Egger, and Prod‐Median), adapted from MR‐Egger and MR‐Median, to enhance the robustness of the MR‐based mediation analysis. We conduct comprehensive simulation studies to compare the existing and proposed methods. The results show that the proposed methods, Diff‐IVW and Prod‐IVW, improve statistical efficiency and type I error control over the existing approaches. Although all IVW‐based methods suffer from directional pleiotropy biases, the median‐based methods (Diff‐Median and Prod‐Median) can mitigate such biases. The differences among the methods can lead to discrepant statistical conclusions as demonstrated in real data applications. Based on our simulation results, we recommend the three proposed methods in practice: Diff‐IVW, Prod‐IVW, and Prod‐Median, which are complementary under various scenarios.

## 1 Introduction

Mediation analysis, increasingly utilized across various research disciplines, aims to assess the transmission of the effect of an exposure (X) on an outcome (Y) through a mediator (M). In mediation analysis, ensuring a causal interpretation of the estimates is an important yet challenging task. Some fairly strong assumptions are usually required to control the exposure‐mediator, exposure‐outcome and mediator‐outcome confounders [1]. However, these assumptions can limit the scope of the applications of mediation analysis. Recently, Mendelian randomization methods (MR) have been applied to address the issue by using genetic variants as the instrumental variables to minimize the confounding effects [2, 3, 4].

We focus on studying MR‐based mediation analysis using summary data, which only requires summary statistics obtained from genome‐wide association studies (GWAS). Most summary‐data MR‐based mediation analyses [5, 6, 7, 8, 9] adopt a procedure akin to the difference or product method described in Burgess et al. [3] (denoted as Diff‐IVW_0_ and Prod‐IVW_0_ hereafter). Burgess et al. [3] suggested Diff‐IVW_0_ as the preferable approach due to inflated type I error in Prod‐IVW_0_. However, Diff‐IVW_0_ assumes null covariance between the estimators of the total and direct effects when deriving the variance of the indirect effect estimator, which may lead to a highly conservative inference [3]. In addition, existing approaches rely on the inverse‐variance weighted method (MR‐IVW) [10], which is known to lack robustness to directional pleiotropy. This study aims to address these issues by presenting two general frameworks for MR‐based causal mediation analysis: the MR‐based difference method and the MR‐based product method. Under the MR‐based difference method, we develop a novel approach Diff‐IVW that accounts for the covariance between the estimators of the total and direct effects when estimating the variances of the mediation effects. Under the MR‐based product method, we propose Prod‐IVW which relies only on univariable MR with rigorous statistical inference. We also adapt pleiotropy‐robust methods from MR‐Egger [11] and MR‐Median [12], and provide robust alternatives for MR‐based mediation analysis. Finally, we conduct comprehensive simulation studies and real data applications to compare the performance of these methods under various scenarios and provide recommendations for future MR‐based mediation studies.

## 2 Method

**FIGURE 1:**
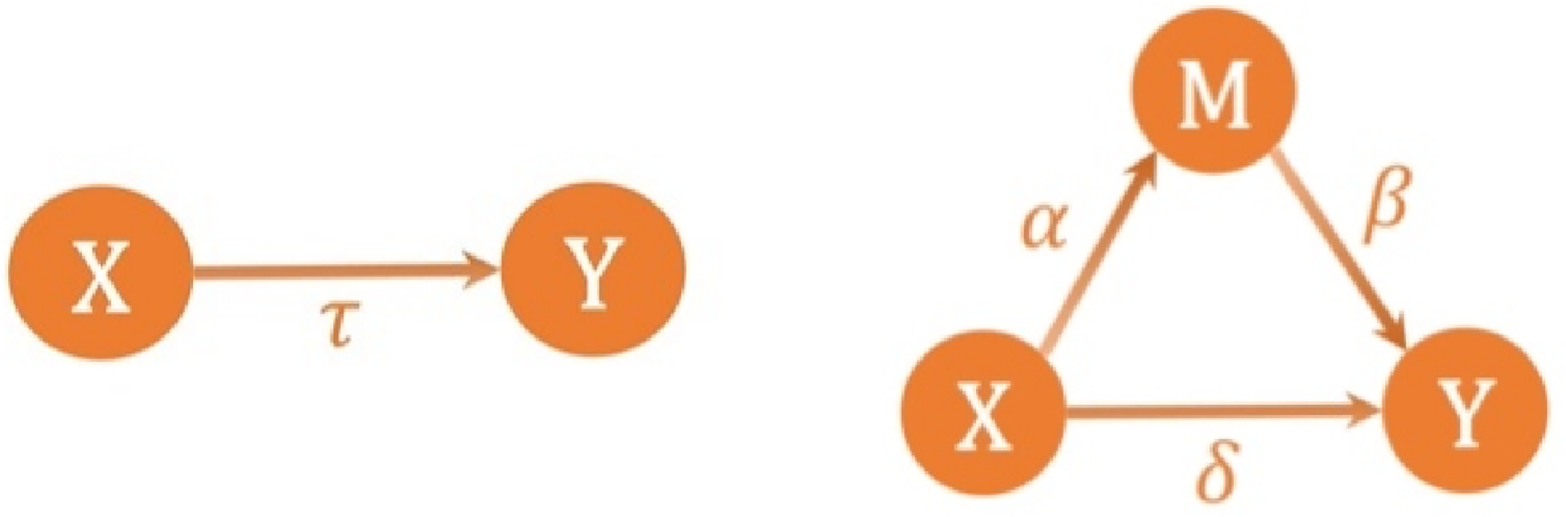
Schematic diagram illustrating the mediation model with a single mediator.

We begin by providing an overview of the single mediator model in linear mediation analysis. Figure 1 depicts the schematic diagram of the mediation model, where τ denotes the total effect of the exposure on the outcome, α denotes the exposure‐mediator effect, β denotes the mediator‐outcome effect when controlling for the exposure, and δ denotes the direct effect of the exposure on the outcome when controlling for the mediator.

Assuming linearity [13, 14], the mediation model can be expressed as:

$$Y=\mu_{1}+\tau X+\gamma_{1}^{T}Z+\epsilon_{1}$$

$$M=\mu_{2}+\alpha X+\gamma_{2}^{T}Z+\epsilon_{2}$$

$$Y=\mu_{3}+\delta X+\beta M+\gamma_{3}^{T}Z+\epsilon_{3},$$

where $\mu_{1}$, $\mu_{2}$, and $\mu_{3}$ are the intercept terms of the linear models, Z represents the observed confounding variable vector with the corresponding vector coefficients $\gamma_{1}$, $\gamma_{2}$, and $\gamma_{3}$ and their transposes are denoted by the superscript *T*, and $\epsilon_{1}$, $\epsilon_{2}$, and $\epsilon_{3}$ are random errors. It follows that τ=αβ+δ, where αβ is the indirect effect of X on Y through M. The core of mediation analysis aims to estimate the total, direct, indirect effects, and the proportion of the total effect mediated by the indirect effect, quantified by ρ=αβ/τ or 1−δ/τ when the direct and indirect effects have the same direction. Linear mediation analysis often applies the product and difference methods to estimate these parameters [15]. However, ensuring a causal interpretation of the estimates through the linear mediation analysis requires certain strong assumptions. These include not only the aforementioned linearity assumptions, but also the consistency assumptions [16, 17] and the absence of unmeasured exposure‐mediator, exposure‐outcome, and mediator‐outcome confounders, as well as no association between the exposure and mediator‐outcome confounders [1, 18]. Recent advancements [19, 20] in causal mediation analysis have broadened the definitions of direct and indirect effects into natural direct effects (NDE) and natural indirect effects (NIE), which do not rely on the linearity assumptions, using the potential outcomes framework. Specifically, let Y(x) denote an individual's potential outcome, possibly contrary to fact, when the exposure X is set to x. The total effect, which contrasts the potential outcomes for an individual when the exposure level changes from x to $x^{\prime }$, is defined as $Y\left(x^{\prime }\right)-Y\left(x\right)$. Similarly, let M(x) represent the potential outcome of the mediator M when the exposure X is set to x, and Y(x,m) denotes the potential outcome when both the exposure X and the mediator M are set to x and m, respectively. The NDE contrasts the potential outcomes for an individual when the exposure level changes from x to $x^{\prime }$, while allowing the mediator to take its natural value under the reference exposure X=x. The NIE contrasts the potential outcomes for an individual under a fixed exposure level $X=x^{\prime }$, but with the mediator taking the value it would naturally assume under different exposure conditions $X=x^{\prime }$ and X=x. Formally, the total effect can be decomposed into the sum of the natural direct effect and natural indirect effect:

$$Y\left(x^{\prime }\right)-Y\left(x\right)=\left[Y\left(x^{\prime },M\left(x^{\prime }\right)\right)-Y\left(x^{\prime },M\left(x\right)\right)\right]+\left[Y\left(x^{\prime },M\left(x\right)\right)-Y\left(x,M\left(x\right)\right)\right]$$

where the first expression on the right‐hand side represents the NIE and the second expression represents the NDE. If we assess the causal effect of a one‐unit change in exposure (i.e., $X=x^{\prime }=x+1$ and X=x) within the linear mediation model, which assumes linearity and no interaction between the exposure and mediator on the outcome, a straightforward calculation yields NDE =δ and NIE =αβ under the aforementioned consistency and no unmeasured confounding assumptions. This demonstrates how NIE and NDE align with the direct and indirect effects defined in the linear mediation model. However, when these stringent parametric assumptions do not hold, it is necessary to use the potential outcomes framework to clearly define the target estimand. Extensive reviews on potential outcomes or counterfactual concepts and recent developments in causal mediation analysis are available in the academic literature [1, 21, 22]. In MR‐based mediation analysis, the assumptions of linearity and no interaction are still required. Therefore, in the following sections, we will refer to the NDE and NIE as the direct and indirect effects, respectively. The key advantage of MR‐based mediation analysis is its ability to address the issue of unmeasured confounders, which will be demonstrated in the subsequent sections.

**FIGURE 2:**
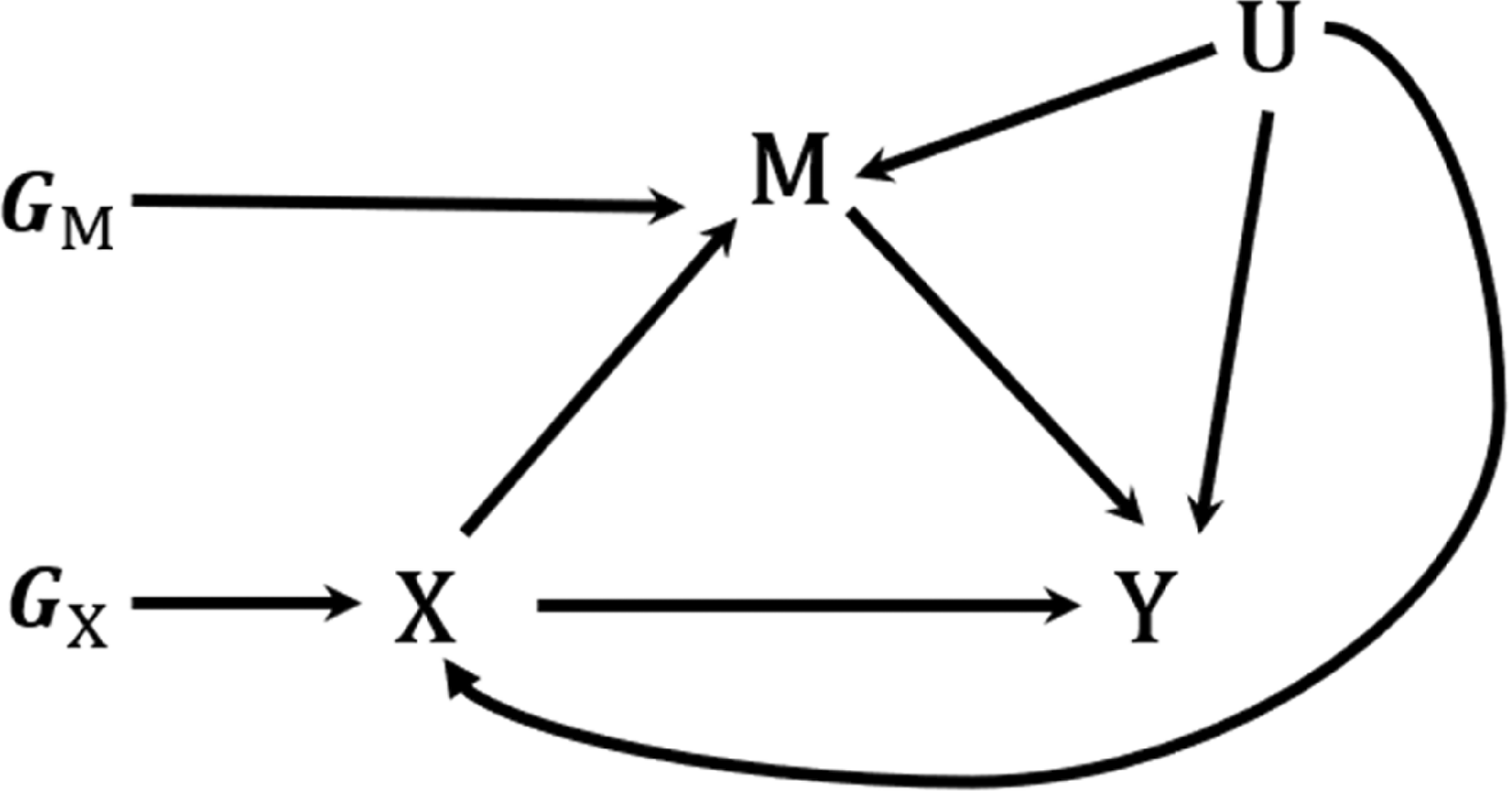
Causal directed acyclic graph (DAG) for Mendelian randomization‐based mediation analysis, depicting the pathway from variable *X* to *Y* through the mediator *M* in a nonparametric structural equation model (NPSEM). It incorporates the genetic instruments *G*_*X*_ and *G*_*M*_, and the unobserved confounders *U*.

Rather than assuming the absence of unmeasured confounders, MR‐based mediation analysis leverages genetic variants (typically single nucleotide polymorphisms [SNPs]) as instrumental variables (IVs) to minimize confounding effects conveyed through a set of confounders denoted as U, presumed to be unobserved in summary‐data MR‐based mediation analysis. For a comprehensive understanding of MR, readers are encouraged to consult MR concepts [23, 24, 25, 26, 27] and recent developments in MR methods [28, 29, 30, 31]. To facilitate MR‐based mediation analysis, two sets of uncorrelated genetic variants are considered as instrumental variables: $G_{X}=\left\{G_{X,1},\cdots ,G_{X,K_{X}}\right\}$ and $G_{M}=\left\{G_{M,1},\cdots ,G_{M,K_{M}}\right\}$, which are associated with X and M, respectively. Figure 2 depicts the causal directed acyclic graph (DAG) illustrating the relationship among the variables in MR‐based mediation analysis. We remark that the genetic instruments $G_{X}$ and $G_{M}$ might not be directly causally related to X and M, respectively; rather, they could serve as proxies for the true causal variants.

**FIGURE 3:**
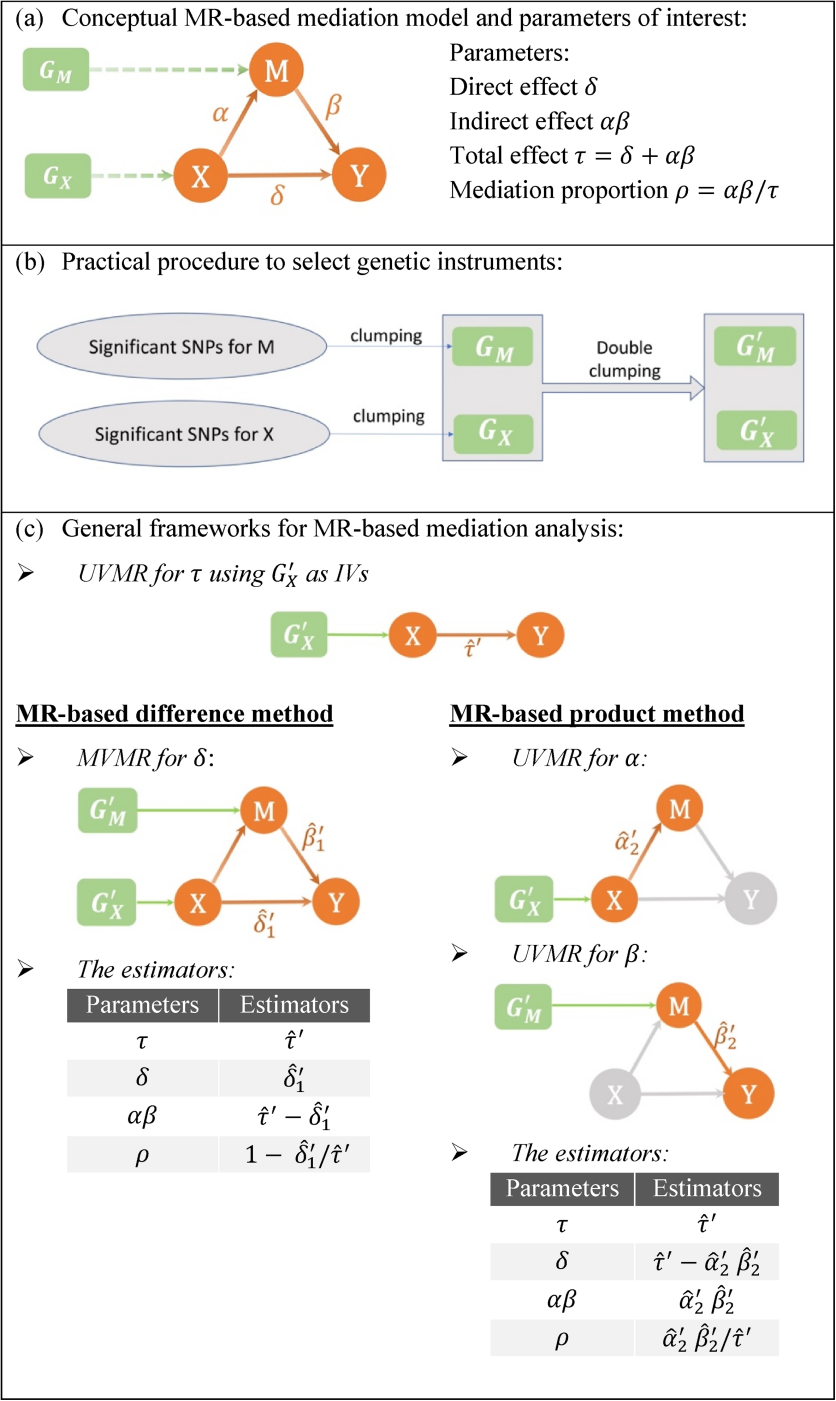
Workflow for the estimation procedures of the proposed MR‐based difference method and MR‐based product method.

By incorporating the genetic variants and maintaining linearity assumptions among the variables, the mediation model is rewritten as follows:

(1) $$X=\mu_{X}+\gamma_{X}^{T}U+\epsilon_{X}+{\;\sum }_{k=1}^{K_{X}}a_{k}G_{X,k}$$

(2) $$M=\mu_{M}+\alpha X+\gamma_{M}^{T}U+\epsilon_{M}+{\;\sum }_{k=1}^{K_{M}}b_{k}G_{M,k}$$

(3) $$Y=\mu_{Y}+\delta X+\beta M+\gamma_{Y}^{T}U+\epsilon_{Y}$$

where $a_{k}$ represents the true association between $G_{X,k}$ and X, $b_{k}$ represents the true association between $G_{M,k}$ and M, $\epsilon_{X}$, $\epsilon_{M}$, and $\epsilon_{Y}$ are random errors, $\mu_{X}$, $\mu_{M}$, and $\mu_{Y}$ are intercept terms, and $\gamma_{X}$, $\gamma_{M}$, and $\gamma_{Y}$ represent the corresponding vector coefficients for U. Despite maintaining the use of α, δ, and β in the MR‐based mediation model, it is important to note that they now denote causal effects, as confounding effects are accounted for in U and its corresponding effects on the variables. Figure 3a illustrates the conceptual MR‐based mediation model and lists the four parameters of interest.

We remark that three assumptions are required for a genetic IV to be valid in MR studies: [10, 25] (i) the IV is associated with the exposure; (ii) the IV is conditionally independent of the outcome given the exposure and confounders (violation of this assumption as discussed in a later section “pleiotropic bias”); and (iii) the IV is independent of unmeasured confounders of the exposure and outcome. It is important to note that in mediation analysis, the mediator plays dual roles for both the outcome and the exposure. We denote two random variables A and B as independent with A⫫B, and A|B means A conditional on B. To provide further clarity, we elaborate on the three IV assumptions within our terminological framework: (i) $a_{k}\neq 0$, (ii) $G_{X,k}$ ⫫ M∣{X,U}, and $G_{X,k}$ ⫫ Y∣{X,U}, and (iii) $G_{X,k}$⫫ U. On the other hand, the three IV assumptions applied to $G_{M,k}$ are (i) $b_{k}\neq 0$ (ii) $G_{M,k}$ ⫫ Y∣{X,M,U}, and (iii) $G_{M,k}$ ⫫ {X,U}. These IV assumptions straightforwardly help to address the unobserved confounders. However, it is worth discussing the presence of mediator‐outcome confounders influenced by the exposure. Burgess et al. [2] discussed these “post‐treatment confounders” and demonstrated the necessity of the linearity assumption without interaction term to ensure well‐defined direct and indirect effects in MR‐based mediation analysis.

### 2.1 Selection of Uncorrelated Genetic Variants

In practice, $G_{X}$ is usually obtained by clumping the genome‐wide significant SNPs for X at a specified threshold for linkage disequilibrium (LD), and a similar clumping procedure is applied to obtain $G_{M}$, as shown in Figure 3b. However, separate clumping of $G_{X}$ and $G_{M}$ does not guarantee the degree of LD when $G_{X}$ and $G_{M}$ are combined. These correlated SNPs may not only affect the accuracy of statistical inferences but also exert potential pleiotropic effects due to their effects on both X and M, which may bias the summary‐data univariable MR (UVMR) estimate for the total effect [4]. A practical strategy is to perform additional clumping on $G_{X}\cup G_{M}$ (termed double clumping in Figure 3b) and assume the mutually uncorrelated subsets $G_{X}^{\prime }\subset G_{X}$ and $G_{M}^{\prime }\subset G_{M}$ were obtained. Without loss of generality, we write $G_{X}^{\prime }\cup G_{M}^{\prime }=\left\{G_{1}^{\prime },\cdots ,G_{K}^{\prime }\right\}$ and symbolize the estimators using $G_{X}^{\prime }$ or $G_{M}^{\prime }$ by the superscription 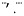 It is important to note that the double clumping procedure preserves the most significant SNP in each LD block, which may still exhibit pleiotropic effects. Ideally, practitioners might opt to eliminate all SNPs exhibiting LD correlation when combining $G_{X}$ and $G_{M}$ as a preferable strategy, despite entailing a trade‐off in power due to the removal of more SNPs. Since pleiotropic effects are typically intricate and difficult to test, we employ pleiotropy‐robust methods to mitigate any residual pleiotropic effects and demonstrate their performance in the simulations.

### 2.2 Total Effect (τ) Through UVMR

Assuming $G_{X,k}$ is a valid IV for the exposure X and outcome Y, we can leverage the relationship $\pi_{k}=\left(\delta +\alpha \beta \right)a_{k}=\tau a_{k}$ to estimate the total effect τ, where $\pi_{k}$ is the true association between $G_{X,k}$ and Y. In practice, we estimate τ using the estimated values of $a_{k}$ and $\pi_{k}$, denoted as ${\hat{a}}_{k}$ and ${\hat{\pi }}_{k}$, respectively, which are typically obtained from summary results of GWAS. Specifically, the widely used MR‐IVW [10] provides an overall estimator by minimizing:

(4) $${\hat{\tau }}_{\mathrm{IVW}}=\underset{\tau \in \mathbb{R}}{\text{argmin}}\sum_{k\in \left\{k|G_{X,k}\in G_{X}\right\}}\frac{{\left({\hat{\pi }}_{k}-\tau \;{\hat{a}}_{k}\right)}^{2}}{{\hat{\sigma }}_{k}^{2}}$$

where ${\hat{\sigma }}_{k}$ is the standard error of ${\hat{\pi }}_{k}$. Although ${\hat{\tau }}_{\mathrm{IVW}}$ is often used to estimate the total effect in the existing MR‐based mediation studies, using the SNPs in $G_{X}/G_{X}^{\prime }$ may bias the total effect estimate as aforementioned. Therefore, we recommend using the subset $G_{X}^{\prime }$ instead of $G_{X}$ in estimating τ:

(5) $${\hat{\tau }}_{\mathrm{IVW}}^{\prime }=\underset{\tau \in \mathbb{R}}{\text{argmin}}\sum_{k\in \left\{k|G_{X,k}\in G_{X}^{\prime }\right\}}\frac{{\left({\hat{\pi }}_{k}-\tau \;{\hat{a}}_{k}\right)}^{2}}{{\hat{\sigma }}_{k}^{2}}$$

To complete the mediation analysis using MR, we estimate the indirect effect, direct effect, and mediation proportion based on the MR‐based difference method and the MR‐based product method as described below.

### 2.3 MR‐Based Difference Method

In linear mediation analysis, the difference method refers to the general procedure of estimating the indirect effect by subtracting the direct effect from the total effect. Given the estimator for the total effect based on UVMR, we can now focus on estimating the direct effect based on multivariable Mendelian randomization (MVMR) [32].

MVMR is an extension of UVMR and can be applied to estimate the direct effects of multiple risk factors on an outcome. Similar to UVMR, the genetic variant used in MVMR should satisfy the IV assumptions [32]. We use $G_{X}^{\prime }\cup G_{M}^{\prime }=\left\{G_{1}^{\prime },\cdots ,G_{K}^{\prime }\right\}$ as the genetic instruments in MVMR and denote the associations between $G_{k}^{\prime }$ and *X*, M and Y by ${\hat{a}}_{k}^{\prime }$, ${\hat{b}}_{k}^{\prime }$ and ${\hat{\pi }}_{k}^{\prime }$, respectively. The estimators of δ and β based on inverse‐variance weighted MVMR (MVMR‐IVW) [33] can be expressed as:

(6) $$\left({\hat{\delta }}_{1,\mathrm{IVW}}^{\prime },{\hat{\beta }}_{1,\mathrm{IVW}}^{\prime }\right)=\underset{\delta ,\beta \in \mathbb{R}}{\text{argmin}}\sum_{k\in \left\{k|G_{k}^{\prime }\in G_{X}^{\prime }\cup G_{M}^{\prime }\right\}}\frac{{\left({\hat{\pi }}_{k}^{\prime }-\delta {\hat{a}}_{k}^{\prime }-\beta {\hat{b}}_{k}^{\prime }\right)}^{2}}{{\left({\hat{\sigma }}_{k}^{\prime }\right)}^{2}},$$

where ${\hat{\sigma }}_{k}^{\prime }$ is the standard error of ${\hat{\pi }}_{k}^{\prime }$. The subscript is utilized to distinguish these estimators from those derived through the MR‐based difference method (subscript 1) and MR‐based product method (subscript 2) as elaborated in the following section. Once the estimator for the direct effect ${\hat{\delta }}_{1,\mathrm{IVW}}^{\prime }$ is obtained, the indirect effect and mediation proportion are being estimated as ${\hat{\tau }}_{\mathrm{IVW}}-{\hat{\delta }}_{1,\mathrm{IVW}}^{\prime }$ and 1 $-{\hat{\delta }}_{1,\mathrm{IVW}}^{\prime }/{\hat{\tau }}_{\mathrm{IVW}}$, respectively. The above existing MR‐based difference estimation procedure is denoted as Diff‐IVW_0_. However, to prevent potential bias caused by SNPs in $G_{X}/G_{X}^{\prime }$ as aforementioned, we propose a revision of Diff‐IVW_0_. Specifically, we replace ${\hat{\tau }}_{\mathrm{IVW}}$ with ${\hat{\tau }}_{\mathrm{IVW}}^{\prime }$ in the estimation procedure. We denote this updated version as Diff‐IVW. We will compare the performance of Diff‐IVW_0_ and Diff‐IVW in the simulation studies and real data applications.

Figure 3c shows the generalized estimation procedure for the MR‐based difference method, which encompasses the IVW‐based and other pleiotropy‐robust methods. In this study, we also consider two alternative methods, namely Diff‐Egger and Diff‐Median. Diff‐Egger employs MR‐Egger [11] to estimate τ and MVMR‐Egger [34] to estimate δ, while Diff‐Median uses MR‐Median [12] to estimate τ and MVMR‐Median [35] to estimate δ. Except for the existing methods, $G_{X}^{\prime }$ will be the default genetic instruments in estimating τ. Table 1 provides a summary of the key differences among the methods.

**TABLE 1:** Summary of the UVMR and MVMR methods applied in the MR‐based mediation methods and the required assumptions.

Method	Estimators via UVMR or MVMR	Key assumptions
Diff‐IVW_0_	τ: MR‐IVW ($G_{X}$) δ: MVMR‐IVW ($G_{X}^{\prime }\cup G_{M}^{\prime }$)	Two‐sample setting. Assume $\mathrm{Cov}\left(\hat{\tau },{\hat{\delta }}_{1}^{\prime }\right)=0$
Diff‐IVW	τ: MR‐IVW ($G_{X}^{\prime }$) δ: MVMR‐IVW ($G_{X}^{\prime }\cup G_{M}^{\prime }$)	Two‐sample setting. Account for $\mathrm{Cov}\left({\hat{\tau }}^{\prime },{\hat{\delta }}_{1}^{\prime }\right)$
Diff‐Egger	τ: MR‐Egger ($G_{X}^{\prime }$) δ: MVMR‐ Egger ($G_{X}^{\prime }\cup G_{M}^{\prime }$)	Two‐sample setting. Account for $\mathrm{Cov}\left({\hat{\tau }}^{\prime },{\hat{\delta }}_{1}^{\prime }\right)$
Diff‐Median	τ: MR‐Median ($G_{X}^{\prime }$) δ: MVMR‐Median ($G_{X}^{\prime }\cup G_{M}^{\prime }$)	Three‐sample setting. Parametric bootstrap
Prod‐IVW_0_	τ: MR‐IVW ($G_{X}$) α: MR‐IVW ($G_{X}^{\prime }$) β: MVMR‐IVW ($G_{X}^{\prime }\cup G_{M}^{\prime }$)	Two‐sample setting
Prod‐IVW	τ: MR‐IVW ($G_{X}^{\prime }$) α: MR‐IVW ($G_{X}^{\prime }$) β: MR‐IVW ($G_{M}^{\prime }$)	Three‐sample setting
Prod‐Egger	τ: MR‐Egger ($G_{X}^{\prime }$) α: MR‐Egger ($G_{X}^{\prime }$) β: MR‐Egger ($G_{M}^{\prime }$)	Three‐sample setting
Prod‐Median	τ: MR‐Median ($G_{X}^{\prime }$) α: MR‐Median ($G_{X}^{\prime }$) β: MR‐Median ($G_{M}^{\prime }$)	Three‐sample setting

*Note*: Two‐sample setting assumes that the samples used to generate summary data of X and M are independent with that of Y. Three‐sample setting assumes samples to generate summary data of X, M, and Y are all independent.

#### 2.3.1 Improved Inference for MR‐Based Difference Method

In this section, our focus is on estimating the variance of the indirect effect estimator, which is crucial for statistical inference regarding mediation effects in MR‐based difference methods. Estimating the indirect effect involves subtracting the estimator for the direct effect from the estimator for the total effect. Therefore, to calculate the variance of the indirect effect, we need to consider the variances of the total and direct effects, as well as their covariance. Although obtaining the variances of the total and direct effects from corresponding MR and MVMR approaches is generally feasible, estimating their covariance can be challenging. To address this issue, Burgess et al. [3] recommended assuming a zero covariance although this may lead to conservative inference.

To estimate the covariance of the total and direct effect estimators in Diff‐IVW, namely $\mathrm{Cov}\left({\hat{\tau }}_{\mathrm{IVW}}^{\prime },{\hat{\delta }}_{1,\mathrm{IVW}}^{\prime }\right)$, we propose a novel approach that leverages the explicit form of the weighted least squares estimates based on MR‐IVW and MVMR‐IVW, and accounts for uncertainty through the heterogeneity parameter based on the multiplicative random effect model. We provide the technical details in Supporting Information: Appendix 1. Once we estimate the covariance, we can compute the variance of the indirect effect for Diff‐IVW as follows:

(7) $$\mathrm{Var}\left({\hat{\tau }}_{\mathrm{IVW}}^{\prime }-{\hat{\delta }}_{1,\mathrm{IVW}}^{\prime }\right)=\mathrm{Var}\left({\hat{\tau }}_{\mathrm{IVW}}^{\prime }\right)+\mathrm{Var}\left({\hat{\delta }}_{1,\mathrm{IVW}}^{\prime }\right)-2\mathrm{Cov}\left({\hat{\tau }}_{\mathrm{IVW}}^{\prime },{\hat{\delta }}_{1,\mathrm{IVW}}^{\prime }\right)$$

Similar procedures can be applied to improve the inference for the mediation proportion and extended for Diff‐Egger. However, the same procedure cannot be directly applied to Diff‐Median because the median‐based estimator lacks explicit formulas. To overcome this difficulty, we generate parametric bootstrap replicates by assuming that the summary‐level statistics of *X*, M, and Y are sampled from independent normal distributions, and adopt the percentile bootstrap intervals for the statistical inference of Diff‐Median. However, this method requires independent samples to produce the summary data of *X*, M, and Y (e.g., three‐sample setting), which may be more challenging to achieve in practice.

### 2.4 MR‐Based Product Method

In mediation analysis, the product method involves estimating the indirect effect αβ by multiplying the estimators of α and β. The existing MR‐based product method, Prod‐IVW_0_, uses MR‐IVW and MVMR‐IVW to estimate α and β, respectively, but may display an inflated type I error when inferring the indirect effect as demonstrated in Burgess et al. [3] To address these limitations, we propose a MR‐based product method that relies solely on UVMR to estimate α and β. This approach closely aligns with the two‐step MR or network MR described in seminal works [2, 4], which primarily focus on individual‐level MR mediation analysis. To ensure rigorous statistical inference for our proposed method, certain assumptions are required. In the following, we provide these assumptions and explain how the delta method can be used for inference.

Assuming $G_{X}^{\prime }$ are valid IVs for estimating the exposure‐mediator effect α, we can use them to perform UVMR and obtain an estimator ${\hat{\alpha }}_{2}^{\prime }$. Similarly, if $G_{M}^{\prime }$ are valid IVs for estimating the mediator‐outcome effect β, we can use them to obtain an MR estimator ${\hat{\beta }}_{2}^{\prime }$. It is important to note that SNPs in $G_{X}/G_{X}^{\prime }$ and $G_{M}/G_{M}^{\prime }$ are excluded when estimating α and β as they may introduce pleiotropic biases. With the estimates ${\hat{\tau }}^{\prime }$, ${\hat{\alpha }}_{2}^{\prime }$, and ${\hat{\beta }}_{2}^{\prime }$, we can estimate the indirect effect by ${\hat{\alpha }}_{2}^{\prime }{\hat{\beta }}_{2}^{\prime }$, the direct effect by ${\hat{\tau }}^{\prime }-{\hat{\alpha }}_{2}^{\prime }{\hat{\beta }}_{2}^{\prime }$, and the mediation proportion by ${\hat{\alpha }}_{2}^{\prime }{\hat{\beta }}_{2}^{\prime }/{\hat{\tau }}^{\prime }$ as shown in Figure 3c.

Note that ${\hat{\tau }}^{\prime }$, ${\hat{\alpha }}_{2}^{\prime }$, and ${\hat{\beta }}_{2}^{\prime }$ are general estimators that can be obtained from any UVMR methods. However, to avoid the bias from the overlapping samples for the UVMR estimates [36], we assume the three‐sample setting. In Supporting Information: Appendix 2, we demonstrate that under certain conditions, ${\hat{\tau }}^{\prime }$, ${\hat{\alpha }}_{2}^{\prime }$, and ${\hat{\beta }}_{2}^{\prime }$ are mutually independent, which enables us to derive the variances of the estimators using the delta method. For example, we can approximate the variance of the estimator ${\hat{\alpha }}_{2}^{\prime }{\hat{\beta }}_{2}^{\prime }$ as.

(8) $$\mathrm{Var}\left({\hat{\alpha }}_{2}^{\prime }{\hat{\beta }}_{2}^{\prime }\right)\approx {\left({\hat{\alpha }}_{2}^{\prime }\right)}^{2}\mathrm{Var}\left({\hat{\beta }}_{2}^{\prime }\right)+{\left({\hat{\beta }}_{2}^{\prime }\right)}^{2}\mathrm{Var}\left({\hat{\alpha }}_{2}^{\prime }\right).$$

The variances for the estimators of δ and ρ can be derived similarly and are provided in Supporting Information: Appendix 2. It is worth noting that the proposed product method is flexible and can adopt any UVMR methods. Specifically, we consider three representative methods: Prod‐IVW, Prod‐Egger, and Prod‐Median. We use MR‐IVW to estimate α, β, and τ in Prod‐IVW, and apply MR‐Egger and MR‐Median for Prod‐Egger and Prod‐Median by analogy as shown in Table 1.

### 2.5 Weak and Pleiotropic Instruments

In the section, we discuss the sources of biases for the MR‐based mediation analysis.

#### 2.5.1 Weak Instrument Bias

In MR, weak instrument bias arises from the violation of the “No Measurement Error” (NOME) assumption for SNP‐exposure associations [37]. In practice, the conventional F‐statistic of the SNP‐association estimate is used to measure the strength of a genetic instrument. Typically, a threshold of *F*‐statistic > 10 is often recommended to address weak instrument bias in UVMR. However, Sanderson et al. [38] indicate that the conventional F‐statistic may not be appropriate to assess the strength of the genetic instruments for MVMR, and propose a two‐sample conditional F‐statistic as the alternative. We will evaluate both the average conventional F‐statistic and two‐sample conditional F‐statistic of the genetic instruments in our simulations and applications.

#### 2.5.2 Pleiotropic Bias

**FIGURE 4:**
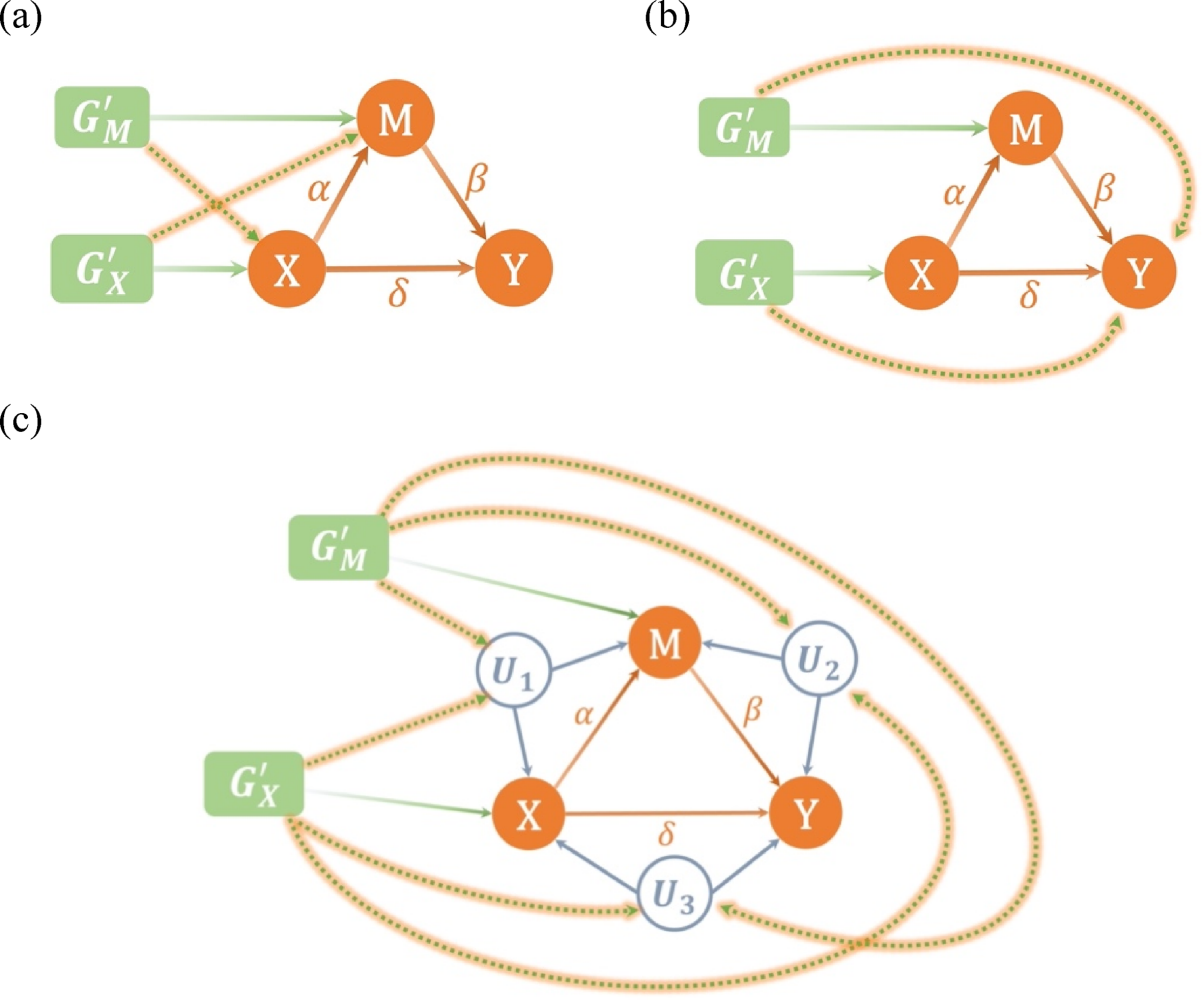
Three types of pleiotropy marked by the dashed lines with orange background: (a) pleiotropy influencing both *X* and *M*; (b) horizontal pleiotropy; (c) confounding pleiotropy, where *U*_1_, *U*_2_, and *U*_3_ represent the exposure‐mediator, mediator‐outcome and exposure‐outcome confounders, respectively.

In genetics, pleiotropy describes the phenomenon of a genetic variation or gene showing effects on multiple traits. In MR literature, pleiotropic bias is thus commonly used to refer to the violation of IV assumption (ii). Pleiotropic bias is known to be a major source of bias in MR‐based estimates. Figure 4 categorizes the pleiotropic effects for the MR‐based mediation analysis in three types: (a) pleiotropy influencing both X and M; (b) horizontal pleiotropy; (c) confounding pleiotropy.

Type (a) pleiotropy, which influences both X and M, may introduce bias in MR‐based mediation analysis, despite allowing for the estimation of the direct effect based on MVMR [32, 33]. For instance, if a genetic instrument of $G_{X}$ is associated with M conditional on X, it is not valid to estimate the total effect as it affects Y without passing through X. On the other hand, if a genetic instrument of $G_{M}$ is associated with X, then it is not a valid IV to estimate the mediator‐outcome effect using UVMR.

To address these pleiotropic effects, we adopt two methods: MR‐Egger and MR‐Median. MR‐Egger is used to address pleiotropy under the Instrument Strength Independent of Direct Effect (InSIDE) assumption [11], and has been extended to MVMR‐Egger [34]. MR‐Median, on the other hand, provides a consistent estimator given that more than half of the genetic instruments are valid [12] and has been extended to MVMR‐Median [35]. We will evaluate the performance of both methods under the three types of pleiotropic effects in the simulation study.

## 3 Simulation

We investigated 11 scenarios to simulate various types of assumption violations as presented in Table 2. For each scenario, we repeated 1000 simulation runs to achieve stable results. We initiated the process by simulating individual‐level data, followed by conducting simple linear regression to estimate the association of X, M, and Y with $G_{X,k}$ and $G_{M,k}$. This procedure yielded the summary‐level data estimates ${\hat{a}}_{k}$, ${\hat{b}}_{k}$ and ${\hat{\pi }}_{k}$.

**TABLE 2:** Summary for the 11 scenarios of the simulations with the three types of pleiotropy, (a)–(c), in Figure 4.

Scenarios	Sample settings	Pleiotropy types	Valid IVs
S1	Two‐sample	No	$G_{X}^{\prime }:60/60,G_{M}^{\prime }:60/60$
S2	Two‐sample	Type (a), balanced	$G_{X}^{\prime }:48/60,G_{M}^{\prime }:48/60$
S3	Two‐sample	Type (b), balanced	$G_{X}^{\prime }:48/60,G_{M}^{\prime }:48/60$
S4	Two‐sample	Type (c), balanced	$G_{X}^{\prime }:48/60,G_{M}^{\prime }:48/60$
S5	Two‐sample	Type (a)–(c), balanced	$G_{X}^{\prime }:34/60,G_{M}^{\prime }:31/60$
S6	Three‐sample	Type (a)–(c), balanced	$G_{X}^{\prime }:34/60,G_{M}^{\prime }:31/60$
S7	Two‐sample	Type (a), directional	$G_{X}^{\prime }:48/60,G_{M}^{\prime }:48/60$
S8	Two‐sample	Type (b), directional	$G_{X}^{\prime }:48/60,G_{M}^{\prime }:48/60$
S9	Two‐sample	Type (c), directional	$G_{X}^{\prime }:48/60,G_{M}^{\prime }:48/60$
S10	Two‐sample	Type (a)–(c), directional	$G_{X}^{\prime }:34/60,G_{M}^{\prime }:31/60$
S11	Three‐sample	Type (a)–(c), directional	$G_{X}^{\prime }:34/60,G_{M}^{\prime }:31/60$

*Note*: Valid IVs: counts for valid IVs out of the total in each scenario.

We set the causal effects as (α,β,δ)= (0.3, 0.3, 0.21) with the mediation proportion ρ=αβ/τ= 0.3. For Scenario 1 (S1), we assumed no pleiotropy, whereas Scenario 2–6 (S2–S6) simulated balanced pleiotropy and Scenario 7–11 (S7–S11) simulated directional pleiotropy. The simulations were mainly conducted under the two‐sample setting, except for S6 and S11 which resembled the pleiotropy in S5 and S10 but assumed the three‐sample setting. To generate the individual‐level data, in S1–S5 and S7–S10, where the two‐sample setting was assumed, we generated 80 000 individuals for the sample of X and M, and independently generated another 80 000 individuals for the sample of Y. In S6 and S11, where the three‐sample setting was assumed, we generated three independent samples for X, M, and Y with 80 000 individuals in each sample.

Specifically, we denote $X_{i}$, $M_{i}$, and $Y_{i}$ as the identically and independently distributed observations of X, M, and Y, respectively, where the subscript i represents the $i^{\mathrm{th}}$ subject in the corresponding samples. Let $G_{X}={\left\{G_{X,k}\right\}}_{1\leq k\leq 80}$ and $G_{M}={\left\{G_{M,k}\right\}}_{1\leq k\leq 80}$ be the collections of genetic variants, and $G_{X}^{\prime }={\left\{G_{X,k}\right\}}_{1\leq k\leq 60}$ and $G_{M}^{\prime }={\left\{G_{M,k}\right\}}_{1\leq k\leq 60}$ be the respective uncorrelated subsets. We denote $G_{X,k,i}$ and $G_{M,k,i}$ as the observations of $G_{X,k}$ and $G_{M,k}$ for the *i*th subjects. We generated $p_{X,k}$ and $p_{M,k}$ from Uniform (0.3, 0.7), where $p_{X,k}$ and $p_{M,k}$ are the allele frequencies for $G_{X,k}$ and $G_{M,k}$, respectively. Specifically, for 1 ≤k≤ 60, $G_{X,k,i}˜\text{Binomial}$ $\left(2,p_{X,k}\right)$ and $G_{M,k,i}˜$ Binomial $\left(2,p_{M,k}\right)$ were independently sampled. For 61 ≤k≤ 80, the pairs of variables, $\left(G_{X,k,i},G_{M,k,i}\right)$, were generated with a correlation 0.5 and the same allele frequency.

Denote $U={\left(U_{1},U_{2},U_{3}\right)}^{T}$ as the unmeasured confounders as shown in Figure 4. We generated $U_{j,i}$ for j= 1, 2, 3 as:

(9) $$U_{j,i}=\epsilon_{j,i}+\sum_{k=1}^{80}d_{X,j,k}G_{X,k,i}+\sum_{k=1}^{80}d_{M,j,k}G_{M,k,i}$$

where ${\left(\epsilon_{1,i},\epsilon_{2,i},\epsilon_{3,i}\right)}^{T}∼\text{Normal}\left(\left[\begin{array}{l} 0\\ 0\\ 0 \end{array}\right],\left[\begin{array}{l} 0.6\\ 0.2\\ 0.4 \end{array}\;\begin{array}{l} 0.2\\ 0.6\\ 0.2 \end{array}\;\begin{array}{l} 0.4\\ 0.2\\ 0.6 \end{array}\right]\right)$, and the coefficients $d_{X,j,k}$ and $d_{M,j,k}$ were zeros except for the scenarios with confounding pleiotropy (S4–S6 and S9–S11) as described later. The individual‐level data was generated by:

(10) $$X_{i}={\gamma_{X}^{T}U+\epsilon_{X,i}+\sum }_{k=1}^{80}a_{X,k}G_{X,k,i}+\sum_{k=1}^{80}a_{M,k}G_{M,k,i}$$

(11) $$M_{i}=\alpha X_{i}+\gamma_{M}^{T}U+\epsilon_{M,i}+{\;\sum }_{k=1}^{80}b_{M,k}G_{M,k,i}+\sum_{k=1}^{80}b_{X,k}G_{X,k,i}$$

(12) $$Y_{i}=\delta X_{i}+\beta M_{i}+\gamma_{Y}^{T}U+\epsilon_{Y,i}+\sum_{k=1}^{80}c_{X,k}G_{X,k,i}+\sum_{k=1}^{80}c_{M,k}G_{M,k,i},$$

where $\gamma_{X}^{T}=$ (1, 0,1), $\gamma_{M}^{T}=$ (1, 1, 0), and $\gamma_{Y}^{T}=$ (0, 1, 1), and $\epsilon_{X,i}$, $\epsilon_{M,i}$, and $\epsilon_{Y,i}$ are random errors distributed as the standard normal distribution.

Next, we provide details for the coefficients' settings of each scenario. The genetic associations $a_{X,k}$ and $b_{M,k}$ were randomly sampled from Uniform (0.02, 0.06). The coefficients to determine the pleiotropy, $\left\{a_{M,k},b_{X,k}\right\}$, $\left\{c_{X,k},c_{M,k}\right\}$, and $\left\{d_{X,j,k},d_{M,j,k}\right\}$, were either zero or randomly sampled from a distribution to simulate the pleiotropic effects as described. For S2, we randomly sampled 20% non‐zero $a_{M,k}$ and $b_{X,k}$ from Uniform (−0.03, 0.03) to simulate the pleiotropy influencing both X and M (type (a) in Figure 4). Similarly, we randomly sampled 20% of non‐zero values $c_{X,k}$ and $c_{M,k}$ from Uniform (−0.03, 0.03) to simulate the horizontal pleiotropy (type (b) in Figure 4) for S3. In S4, we simulated the confounding pleiotropy (type (c) in Figure 4). We randomly selected 20% of SNPs and generated non‐zero values of $d_{X,j,k}$ for 1 ≤j≤ 3 at the selected SNPs from Uniform (−0.03, 0.03). A similar procedure was applied to generate $d_{M,j,k}$. Scenario 5 mixed all the pleiotropy effects from S2 to S4 together. S6 resembled S5 but assumed the three‐sample setting as mentioned. Note that the pleiotropic effects of S2–S6 were balanced in the sense that the coefficients for the pleiotropy were sampled from the zero‐mean distribution, Uniform (−0.03, 0.03). We modified the balanced pleiotropy to the directional pleiotropy in S7–S11 by analogy to S2–S6. Specifically, we changed the distribution to generate non‐zero coefficients of $a_{M,k}$, $b_{X,k}$ $c_{X,k}$, $c_{M,k}$, $d_{X,j,k}$, and $d_{M,j,k}$ from Uniform (−0.03, 0.03) to Uniform (−0.02, 0.04). Under this setting, the genetic variants $G_{X}^{\prime }$ and $G_{M}^{\prime }$ explained approximately 3.3% and 2.6% of the variances of X and M in S1, respectively. Moreover, the average F‐statistic was around 45.0 and 36.1 for $G_{X}^{\prime }$ and $G_{M}^{\prime },$ respectively, while the two‐sample conditional F‐statistic was approximately 18.8 and 16.9. The other scenarios shared similar statistics regarding the explained variance and the average F‐statistic.

In Supporting Information: Appendix 3, we conducted additional simulations using binary outcomes generated from a logistic regression model following the same estimating procedures. This model is known to demonstrate non‐collapsibility of odds ratios, which may impact the MR‐based mediation analysis. Nevertheless, our simulation results show that the influence of non‐collapsibility is not substantial under the rare outcome assumption (usually < 10% prevalence) [39, 40, 41]. This confirms the robustness of our analysis under this assumption. Importantly, we emphasize that the proposed summary‐data MR‐based mediation approaches should be regarded as approximations in the case of binary outcomes, even under the rare outcome assumption. In general, estimators based on product and difference methods are biased in binary outcome cases without additional assumptions and constraints, as noted in the literature [42, 43].

### 3.1 Simulation Results

To evaluate the accuracy of the point estimates, we compare the mean squared errors (MSEs) for the total effect (TE), direct effect (DE), indirect effect (IE), and mediation proportion (ρ).

**FIGURE 5:**
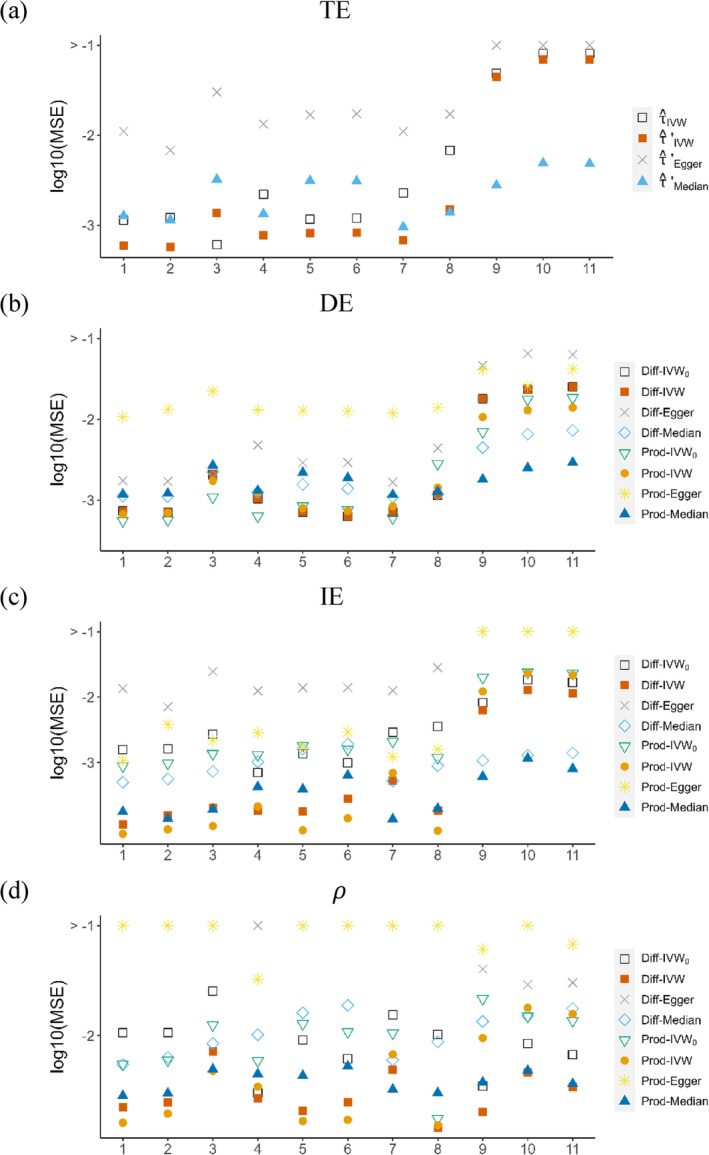
Logarithm of the mean squared errors (MSEs) for (a) total effect (TE), (b) direct effect (DE), (c) indirect effect (IE), and (d) mediation proportion (ρ). Lower points on the graph indicate better accuracy.

In Figure 5a, we present the results for TE and note that ${\hat{\tau }}_{\mathrm{IVW}}^{\prime }$ outperformed the alternative methods in S1–S6 but showed a marked increase in S7–S11 when the pleiotropic effects were directional. Although more genetic instruments were used in ${\hat{\tau }}_{\mathrm{IVW}}$, the additional SNPs in $G_{X}/G_{X}^{\prime }$ did not provide evident benefits as they might bias the estimates of TE. On the other hand, the median‐based estimate, ${\hat{\tau }}_{\text{Median}}$, was more robust to the directional pleiotropy in S7–S11 while the Egger‐based estimate, ${\hat{\tau }}_{\text{Egger}}$, had high MSEs even in S3 and S8 where the pleiotropy satisfied the InSIDE assumption. Similar findings were observed for DE as shown in Figure 5b, where the IVW‐based method performed well in scenarios with no or balanced pleiotropy and the median‐based method provided better alternatives in scenarios with directional pleiotropy. Note that Diff‐IVW_0_ and Diff‐IVW overlapped in Figure 5b as they employed the same approach in estimating DE.

Regarding IE in Figure 5c, the IVW‐based methods, Diff‐IVW and Prod‐IVW, outperformed the other methods in S1–S6. However, they showed a marked increase in MSEs in S9–S11, where extensive directional pleiotropy was present. In contrast, the two median‐based methods (Diff‐Median and Prod‐Median) performed well in these scenarios.

Comparing the MSEs of ρ is more complicated due to its ratio type estimators (Figure 5d). For example, the MR‐based difference method estimates ρ as ${\hat{\rho }}_{1}^{\prime }=1-{\hat{\delta }}_{1}^{\prime }/{\hat{\tau }}^{\prime }.$ If ${\hat{\delta }}_{1}^{\prime }$ and ${\hat{\tau }}^{\prime }$ are biased towards the same direction, the bias of ${\hat{\rho }}_{1}^{\prime }$ may not be as strong as that of ${\hat{\delta }}_{1}^{\prime }$ or ${\hat{\tau }}^{\prime }$ since the bias could be partially canceled out. This might explain the relatively moderate increases in the MSEs of ρ in scenarios with directional pleiotropy.

Based on the MSEs, we conclude that the IVW‐based methods perform well in scenarios with no or balanced pleiotropy, while the median‐based methods provide robust alternatives when extensive directional pleiotropy exists. However, we do not recommend the Egger‐based methods (Diff‐Egger and Prod‐Egger) due to their consistently high level MSEs across all scenarios.

**FIGURE 6:**
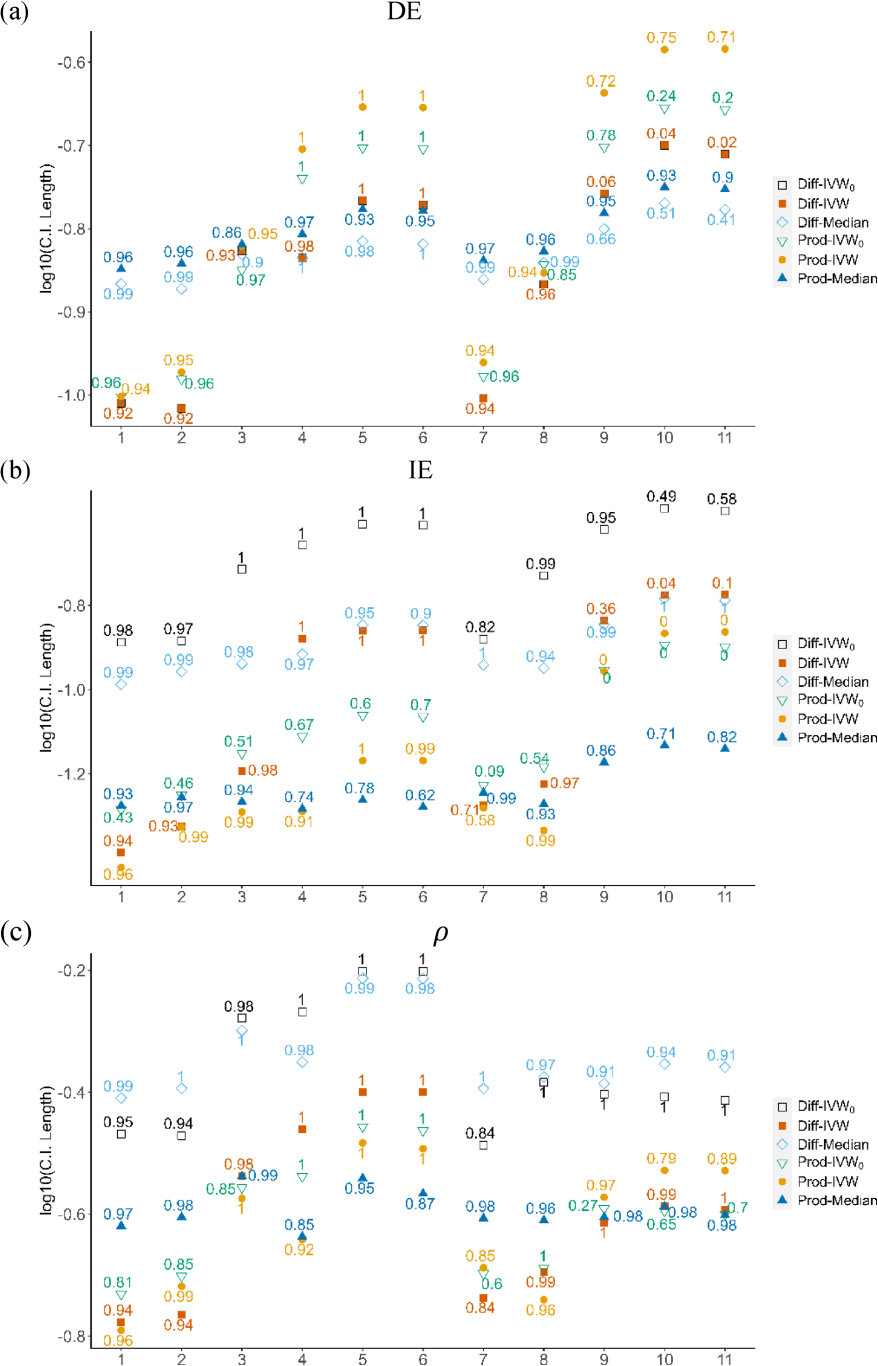
Logarithm of the interval lengths and empirical coverage rates (shown in number) of the 95% confidence intervals for (a) direct effect (DE), (b) indirect effect (IE), and (c) mediation proportion (ρ).

Next, we compare the lengths (efficiency) and empirical coverage rates (accuracy) of the 95% CIs for DE, IE, and ρ across the methods. We used 1000 bootstrap replicates for the statistical inference of Diff‐Median. The results are summarized in Figure 6 and all the numerical results are provided in Supporting Information: Appendix 5. For simplicity, we omitted the results for TE and Egger‐based methods in the figure. Since Diff‐IVW_0_ and Diff‐IVW had the same estimation for DE, they were perfectly aligned in Figure 6a. However, there existed visible differences between the two methods for IE and ρ as shown in Figure 6b,c. For IE, Diff‐IVW_0_ had the widest CIs with coverage rates higher than the confidence level across in S1–S6. This indicated that Diff‐IVW_0_ was too conservative for inferring IE. In contrast, Diff‐IVW provided smaller CIs for IE and ρ with satisfactory coverage rates in S1–S6 (0.93–1 for IE and 0.94–1 for ρ). On the other hand, Prod‐IVW performed well in S1–S6 while Prod‐IVW_0_ displayed inflated type I error with the coverage rate 0.43 for IE in S1 where no pleiotropy was present. However, the coverage rates for IE of Diff‐IVW and Prod‐IVW dropped to nearly zero in S9–S11 as the directional pleiotropy added up. This indicated that the statistical inferences for IE based on these IVW‐based methods were no longer valid when extensive directional pleiotropy existed.

In contrast to the IVW‐based methods, the median‐based methods (Diff‐Median and Prod‐Median) maintained coverage rates away from zero in all scenarios. This finding aligns with the MSE results and suggests that the median‐based methods are more robust to directional pleiotropy than the IVW‐based methods. However, the coverage rates for the median‐based methods might not reach the nominal level in S1–S6, where there existed no or balanced pleiotropy (while maintaining a stable interval length). Overall, the IVW‐based methods and the median‐based methods complement each other in different types of pleiotropy.

Interestingly, the MR‐based product method performed well in S1–S5 where the two‐sample setting was assumed. In these scenarios, UVMR was used to estimate the exposure‐mediator effect based on summary‐level data from a single sample and no significant difference was found when comparing S5 and S10 (two‐sample setting) to S6 and S11 (three‐sample setting). However, we should note that the overlapping sample can bias the UVMR estimates [36] (see Section 5).

In summary, we recommend using three methods in practice: Diff‐IVW, Prod‐IVW, and Prod‐Median because they offer better efficiency and accuracy, and their complementary performance in different scenarios.

## 4 Investigate Potential Mediators Between Waist‐To‐Hip Ratio and Cardiovascular Disease

To demonstrate the methods described above, we revisit the examples studied by Gill et al. [5], which investigated the potential mediators between waist‐to‐hip ratio (WHR) as the exposure and cardiovascular disease (CAD) as the outcome, to compare our methods to previous methods in a real data analysis setting. In Gill et al., both smoking behaviors (SMK) and type 2 diabetes (T2D) were investigated as potential mediators. While T2D showed a substantial mediation proportion of 30%, it was not statistically significant (95% CI from −3% to 63%) [5] (Note that a negative mediation proportion may lack appropriate interpretations). Utilizing our proposed method, we demonstrate that T2D indeed showed a significant mediation proportion for the effect of WHR on CAD (Table 3). On the other hand, SMK exhibited a modest 10% mediation proportion (95% CI from −24% to 45%) [5]. Our approach showed results with similar non‐significant mediation proportion estimates (Table 4). We used the same publicly available GWAS summary data examined in Gill et al. [5] Readers are referred to the corresponding literatures [44, 45, 46, 47] and Supporting Information: Appendix 4 for more detailed information of the data.

**TABLE 3:** MR‐based mediation analysis for WHR (exposure), T2D (mediator), and CAD (outcome).

Method	TE	DE	IE	ρ
Diff‐IVW_0_	1.57 (1.41, 1.75)	1.38 (1.22, 1.56)	1.14 (0.97, 1.34)	0.284 (−0.036, 0.604)
Diff‐IVW	1.56 (1.39, 1.76)	1.38 (1.22, 1.56)	1.13 (1.07, 1.2)	0.273 (0.136, 0.41)
Diff‐Egger	1.41 (1.01, 1.96)	1.3 (1.04, 1.62)	1.09 (0.85, 1.38)	0.243 (−0.315, 0.8)
Diff‐Median	1.52 (1.34, 1.77)	1.43 (1.22, 1.6)	1.06 (0.99, 1.22)	0.133 (−0.028, 0.459)
Prod‐IVW_0_	1.57 (1.41, 1.75)	1.36 (1.21, 1.53)	1.16 (1.1, 1.21)	0.323 (0.192, 0.454)
Prod‐IVW	1.56 (1.39, 1.76)	1.36 (1.19, 1.54)	1.15 (1.1, 1.21)	0.318 (0.18, 0.456)
Prod‐Egger	1.41 (1.01, 1.96)	1.39 (1, 1.95)	1.01 (0.94, 1.08)	0.029 (−0.18, 0.238)
Prod‐Median	1.52 (1.32, 1.74)	1.36 (1.17, 1.58)	1.11 (1.05, 1.18)	0.257 (0.087, 0.428)

*Note*: The odds ratios of TE, DE and IE and the mediation proportion are provided with 95% confidence intervals.

Abbreviations: DE = direct effect; IE = indirect effect; TE = total effect, and ρ is the mediation proportion.

**TABLE 4:** MR‐based mediation analysis for WHR (exposure), SMK (mediator), and CAD (outcome).

Method	TE	DE	IE	ρ
Diff‐IVW_0_	1.57 (1.41, 1.75)	1.54 (1.37, 1.73)	1.02 (0.87, 1.2)	0.046 (−0.294, 0.387)
Diff‐IVW	1.58 (1.41, 1.77)	1.54 (1.37, 1.73)	1.03 (0.99, 1.07)	0.059 (−0.025, 0.142)
Diff‐Egger	1.39 (1.01, 1.93)	1.57 (1.21, 2.05)	0.89 (0.73, 1.07)	−0.368 (−1.204, 0.468)
Diff‐Median	1.58 (1.33, 1.74)	1.65 (1.38, 1.79)	0.96 (0.89, 1.05)	−0.091 (−0.318, 0.112)
Prod‐IVW_0_	1.57 (1.41, 1.75)	1.53 (1.37, 1.71)	1.03 (0.99, 1.06)	0.057 (−0.013, 0.128)
Prod‐IVW	1.58 (1.41, 1.77)	1.54 (1.37, 1.73)	1.03 (1, 1.06)	0.065 (0, 0.129)
Prod‐Egger	1.39 (1.01, 1.93)	1.38 (1, 1.92)	1.01 (0.96, 1.05)	0.016 (−0.113, 0.144)
Prod‐Median	1.58 (1.39, 1.8)	1.55 (1.35, 1.76)	1.02 (1, 1.05)	0.051 (−0.001, 0.103)

*Note*: The odds ratios of TE, DE and IE, and the mediation proportion are provided with 95% confidence intervals.

Abbreviations: DE = direct effect; IE = indirect effect; TE = total effect, and ρ is the mediation proportion.

Table 3 reveals notable inconsistencies in assessing the mediating effect of T2D. Although the Diff‐IVW_0_, Diff‐Egger, Diff‐Median, and Prod‐Egger methods do not show a statistically significant difference between the mediating effect of T2D and zero, the Diff‐IVW, Prod‐IVW_0_, Prod‐IVW, and Prod‐Median methods demonstrate a statistically significant mediating effect at a 95% confidence level. Specifically, the estimated mediation proportion by Diff‐IVW_0_ is 28.4% (95% CI from −3.6% to 60.4%), which is comparable to the mediation proportion in Gill et al. [5]. However, the three recommended methods estimate the mediation proportion as: 27.3% (95% CI from 13.6% to 41%) by Diff‐IVW, 31.8% (95% CI from 18% to 45.6%) by Prod‐IVW, and 25.7% (95% CI from 8.7% to 42.8%) by Prod‐Median. The discrepant conclusions across the methods may be attributed to the differences in their efficiency. Based on the three recommended methods: Diff‐IVW, Prod‐IVW, and Prod‐Median, we conclude that T2D significantly mediates the effect of WHR on CAD.

We note that the summary data for WHR and T2D were derived from GWAS meta‐analyses and contained overlapping samples from UK Biobank. To strengthen our findings, we conducted a sensitivity analysis in a three‐sample setting by utilizing an earlier GWAS of T2D that did not include samples from UK Biobank. The results of this analysis also support the conclusion that T2D significantly mediates the effect of WHR on CAD. Additional details regarding this analysis can be found in Supporting Information: Appendix 4.

Table 4 provides the MR‐based mediation estimates for WHR (exposure), SMK (mediator), and CAD (outcome), with TE, DE and IE estimates converted into odds ratios. The 95% CIs for IE and ρ cover the null or boundary near null, indicating that the mediation effect of SMK is not significant or only marginally significant. These results suggest that SMK does not play a significant role in mediating the effect of WHR on CAD. Specifically, Diff‐IVW_0_ estimates the mediation proportion as 4.6% (95% CI from −29.4% to 38.7%), which is comparable to the mediation proportion in Gill et al. [5] However, the 95% CI for the mediation proportion based on Diff‐IVW_0_ is approximately four times wider than the 95% CI (from −2.5% to 14.2%) based on Diff‐IVW. This discrepancy is consistent with our simulation study, which demonstrated that Diff‐IVW_0_ is less efficient than Diff‐IVW due to ignoring the covariance between the estimators of the total and direct effects.

## 5 Discussion

In this study, we have presented two summary‐data MR‐based frameworks for causal mediation analysis and proposed various MR‐based mediation methods under these frameworks. Notably, we develop Diff‐IVW, which improves upon the existing MR‐based difference method by accounting for the covariance between the estimators of the total effect and direct effect when estimating the mediation effects. Furthermore, we propose the MR‐based product method, which relies solely on UVMR and has a rigorous statistical inference framework. Based on our simulation results, we recommend three methods for practical use: Diff‐IVW, Prod‐IVW and Prod‐Median.

There are pros and cons to both the MR‐based difference method and MR‐based product method. The MR‐based difference method can potentially perform mediation analysis with multiple mediators but requires an extended clumping procedure for multiple traits and MVMR, which may be susceptible to weak instrument bias. On the other hand, the MR‐based product method is more flexible as it can accommodate any UVMR method. However, generalizing it to multiple mediators can be challenging [4]. Additionally, the three‐sample setting required by the product method may be difficult to achieve in practice. However, a recent study [48] has shown that MR‐IVW and MR‐Median perform well when using one‐sample summary‐level data derived from a single large dataset, such as UK Biobank data. This finding aligns with our simulation results, where Prod‐IVW and Prod‐Median performed well in the two‐sample setting. This opens up the possibility of using the MR‐based product method when there are overlapping samples between the exposure and mediator.

Although Mendelian randomization can extend the scope of linear mediation analysis, one of the main concerns is directional pleiotropy as demonstrated in our simulations. To address this concern, we investigated the Egger‐based and median‐based methods as alternatives and concluded that the median‐based methods are more robust to directional pleiotropy. In real data applications, pleiotropic effects are often unknown and can be complex. Therefore, it is important to implement pleiotropy‐robust methods in addition to IVW‐based methods to strengthen the evidence.

However, it is crucial to acknowledge that MR‐based mediation analysis relies on certain strong instrumental variable assumptions, which are typically untestable in practice. Moreover, the proposed MR‐based mediation analysis also necessitates linearity assumptions for the effects of exposure, mediator, and outcome, and violations of linearity can introduce bias. Researchers should proceed with caution when applying MR‐based mediation analysis. Although addressing this is beyond the scope of the study, future work is encouraged to explore more flexible MR‐based mediation approaches.

In summary, our proposed methods improve upon the efficiency and accuracy of existing approaches and provide pleiotropy‐robust alternatives to enhance the robustness of summary‐data MR‐based mediation analysis.

## Author Contributions

Shu‐Chin Lin designed the statistical method, performed data analysis and drafted the manuscript. Chia‐Yen Chen conceived the idea, devised and supervised the study. Yen‐Feng Lin conceived the idea, supervised and acquired funding for the study. All authors revised and approved the final manuscript.

## Disclosure

Software: R codes for implementing the MR‐based mediation methods and for reproducing the results in the study are available on GitHub: https://github.com/scllin/mrMed. Clumping of correlated SNPs was performed using the R package: TwoSampleMR (https://github.com/mrcieu/TwoSampleMR). The two‐sample conditional F‐statistic was computed based on the R package: MVMR (https://github.com/WSpiller/MVMR).

## Conflicts of Interest

Chia‐Yen Chen is an employee of Biogen. All other authors declare no conflicts of interest.

## Supporting information


**Data S1.** Supporting Information.

## Data Availability

The WHR GWAS data is available from the GIANT consortium (https://portals.broadinstitute.org/collaboration/giant/index.php/GIANT_consortium_data_files). The CAD GWAS data is available from the CARDIoGRAMplusC4D consortium (http://www.cardiogramplusc4d.org/). The T2D GWAS data is available from the DIAGRAM consortium (http://diagram‐consortium.org/). Lifetime smoking GWAS data is available for download at https://doi.org/10.5523/bris.10i96zb8gm0j81yz0q6ztei23d.
