# Supplementary material for: Causal Mediation Analysis: A Summary‐Data Mendelian Randomization Approach
Source: Stat Med. 2025 Feb 5;44(5):e10317. doi: 10.1002/sim.10317 (PMC11799828; doi:10.1002/sim.10317)
Supplement: Supplementary file 1 — Data S1. Supporting Information. [file SIM-44-0-s001.zip › supplement_mrMed_r2_241029.docx]

**Supplement for “Causal Mediation Analysis: A Summary-Data Mendelian Randomization Approach”**

Shu-Chin Lin^1^, Sheng-Hsuan Lin^2^, Tian Ge^3,4,5^, Chia-Yen Chen^6*^, Yen-Feng Lin^1,7,8*^

^1^Center for Neuropsychiatric Research, National Health Research Institutes, Miaoli, Taiwan

^2^Institute of Statistics, National Yang Ming Chiao Tung University, Hsinchu, Taiwan.

^3^Psychiatric and Neurodevelopmental Genetics Unit, Center for Genomic Medicine, Massachusetts General Hospital, Boston, MA, USA

^4^Stanley Center for Psychiatric Research, Broad Institute of MIT and Harvard, Cambridge, MA, USA

^5^Department of Psychiatry, Massachusetts General Hospital, Harvard Medical School, Boston, MA, USA

^6^Biogen, Cambridge, MA, USA

^7^Department of Public Health & Medical Humanities, School of Medicine, National Yang Ming Chiao Tung University, Taipei, Taiwan

^8^Institute of Behavioral Medicine, College of Medicine, National Cheng Kung University, Tainan, Taiwan

*Corresponding author

# Appendix 1. Statistical inference for Diff-IVW

In the section, we propose an estimator for $Cov\left( \hat{\tau}_{IVW}^{'},\hat{\delta}_{1, IVW}^{'} \right)$, the covariance between the estimators of the total effect and direct effect, to complete the statistical inference of Diff-IVW.

Recall that $G_{X}^{'}\cup G_{M}^{'}=\{G_{1}^{'},\cdots,G_{K}^{'}\}$ are the genetic instruments selected by the double clumping procedure. We denote the associations between $G_{k}^{'}$ and *X*, $M$ and $Y$ by $\hat{a}_{k}^{'}$*,* $\hat{b}_{k}^{'}$ and $\hat{\pi}_{k}^{'}$, respectively. Let

|  | $\Pi=\left[ \begin{matrix} \hat{\pi}_{1}^{'} & \cdots& \hat{\pi}_{K}^{'} \end{matrix} \right]^{T}$, | (1) |
| --- | --- | --- |
|  | $\Gamma=\left[ \begin{matrix} \hat{a}_{1}^{'} & \cdots& \hat{a}_{K}^{'} \\ \hat{b}_{1}^{'} & \cdots& \hat{b}_{K}^{'} \end{matrix} \right]^{T}$, | (2) |

and $W$ be the *K*-by-*K* diagonal matrix whose $k^{th}$ diagonal element is ${(\sigma_{k}^{'})}^{2}$, the variance of $\hat{\pi}_{k}^{'}$. The estimators $\hat{\delta}_{1,IVW}^{'}$ and $\hat{\beta}_{1,IVW}^{'}$ (see Equation (6) of the main paper) have the explicit form:

|  | $\left[ \begin{matrix} \hat{\delta}_{1,IVW}^{'} \\ \hat{\beta}_{1,IVW}^{'} \end{matrix} \right]=\left( \Gamma^{T}W^{-1}\Gamma\right)^{-1}\Gamma^{T}W^{-1}\Pi$ | (3) |
| --- | --- | --- |

based on weighted least squares.

Next, we consider the explicit form of $\hat{\tau}_{IVW}^{'}$ as defined in Equation (5) of the main paper. Without loss of generality, we assume $G_{X}^{'}=\{G_{1}^{'},\cdots G_{s}^{'}\}$ and $G_{M}^{'}=\{G_{s+1}^{'},\cdots G_{K}^{'}\}$. Let $\Lambda=\left[ \begin{matrix} \hat{a}_{1}^{'} & \cdots& \hat{a}_{s}^{'} \end{matrix} \right]^{T}$, $\Pi_{s}=\left[ \begin{matrix} \hat{\pi}_{1}^{'} & \cdots& \hat{\pi}_{s}^{'} \end{matrix} \right]^{T}$ and $W_{s}$ be the corresponding $S$-by-$S$ matrix whose $s^{th}$ diagonal element is ($\sigma_{s}^{'}$)^2^, then we have

|  | $\hat{\tau}_{IVW}^{'}=\left( \Lambda^{T}W_{s}^{-1}\Lambda\right)^{-1}\Lambda^{T}W_{s}^{-1}\Pi_{s}$*.* | (4) |
| --- | --- | --- |

Under the two-sample setting, $\Pi$ is independent of $\Gamma$. Therefore, given $\Gamma$, we have

| $Cov\left( \left[ \begin{matrix} \hat{\delta}_{1,IVW}^{'} \\ \hat{\beta}_{1,IVW}^{'} \end{matrix} \right],\hat{\tau}_{IVW}^{'} \right)$  $=\left( \Gamma^{T}W^{-1}\Gamma\right)^{-1}\Gamma^{T}W^{-1}Cov\left( \Pi,\Pi_{s}^{T} \right)W_{s}^{-1}\Lambda\left( \Lambda^{T}W_{s}^{-1}\Lambda\right)^{-1}.$ | (5) |
| --- | --- |

We only need to estimate $Cov\left( \Pi,\Pi_{s}^{T} \right)$. Based on the multiplicative random-effects model,[^1^](#_ENREF_1) it is not hard to derive $Cov\left( \Pi,\Pi_{s}^{T} \right)=\phi^{2} W(K,S)$, where $W(K,S)$ is the submatrix corresponding to the first *K* rows and *S* columns of $W$ and $\phi$ is the heterogeneity parameter[^1^](#_ENREF_1). The heterogeneity parameter, $\phi$, is usually estimated by the residual standard error of the regression. Specifically, we can either estimate $\phi$ as the residual standard error, $\hat{\phi}_{1}$, from Equation (5) of the main paper, or as the residual standard error, $\hat{\phi}_{2}$, from Equation (6) of the main paper. For the sake of conservativity, we recommend estimating $\phi$ by $\hat{\phi}=min\left( \hat{\phi}_{1},\hat{\phi}_{2} \right)$ to avoid underestimating the variance for the indirect effect. In addition, we suggest setting $\hat{\phi}\geq1$, as suggested by previous studies,[^1^](#_ENREF_1)^,^[^2^](#_ENREF_2) to correct for cases of underdispersion. We also use the Cauchy–Schwarz inequality to set the upper bound $Cov\left( \hat{\tau}_{IVW}^{'},\hat{\delta}_{1,IVW}^{'} \right)\leq\{Var\left( \hat{\tau}_{IVW}^{'} \right)Var{\left( \hat{\delta}_{1, IVW}^{'} \right)\}}^{1/2}$ . Thus, we can estimate the variance for the indirect effect estimator by

| $Var\left( \hat{\tau}_{IVW}^{'}-\hat{\delta}_{1,IVW}^{'} \right)=Var\left( \hat{\tau}_{IVW}^{'} \right)+Var\left( \hat{\delta}_{1, IVW}^{'} \right)-2Cov\left( \hat{\tau}_{IVW}^{'},\hat{\delta}_{1,IVW}^{'} \right).$ | (6) |
| --- | --- |

On the other hand, the variance for the mediation proportion can be approximated by

| $Var\left( 1-\frac{\hat{\delta}_{1,IVW}^{'}}{\hat{\tau}_{IVW}^{'}} \right)$  $\approx\frac{Var\left( \hat{\delta}_{1,IVW}^{'} \right)}{{(\hat{\tau}_{IVW}^{'})}^{2}}+\frac{\left( \hat{\delta}_{1,IVW}^{'} \right)^{2}Var\left( \hat{\tau}_{IVW}^{'} \right)}{({\hat{\tau}_{IVW}^{'})}^{4}}-2\frac{\hat{\delta}_{1,IVW}^{'}Cov\left( {\hat{\tau}_{IVW}^{'},\hat{\delta}}_{1,IVW} \right)}{{(\hat{\tau}_{IVW}^{'})}^{3}}$ | (7) |
| --- | --- |

based on the delta method[^3^](#_ENREF_3) assuming asymptotic normality of the MR-based estimates. All the components on the right-hand side have been well-estimated. This completes the statistical inference for Diff-IVW. We note that similar procedure can be applied to develop the statistical inference for Diff-Egger and we omit the details.

# Appendix 2. Statistical inference for MR-based product method

We provide theoretical support and develop the variances of the direct effect and mediation proportion for the MR-based product method.

In MR studies, it is common to assume no measurement error of the estimated genetic association, which is known as the no measurement error assumption (NOME).[^4^](#_ENREF_4) If NOME holds for the $G_{X}^{'}$-exposure and $G_{M}^{'}$-mediator associations, then $\hat{\alpha}_{2}^{'}$ and $\hat{\beta}_{2}^{'}$ are independent since the summary data of $M$ and $Y$ are independent under the three-sample setting. Similar arguments can be applied to show the independence between $\hat{\alpha}_{2}^{'}$ and $\hat{\tau}'$. On the other hand, note that $\hat{\tau}'$ and $\hat{\beta}_{2}^{'}$ depend on the information carried by $G_{X}^{'}$ and $G_{M}^{'}$, respectively. Following,[^5^](#_ENREF_5) we assume that the uncorrelated genetic instruments $G_{X}^{'}$ and $G_{M}^{'}$ provide independent information on the corresponding estimates, so that $\hat{\tau}'$ and $\hat{\beta}_{2}^{'}$ are independent. In sum, $\hat{\tau}'$, $\hat{\alpha}_{2}^{'}$, and $\hat{\beta}_{2}^{'}$ are mutually independent under the above assumptions. Therefore, the variances of the proposed estimators can be calculated based on the delta method:

|  | $Var\left( \hat{\alpha}_{2}^{'}\hat{\beta}_{2}^{'} \right)$ ≈ $\left( \hat{\alpha}_{2}^{'} \right)^{2}Var\left( \hat{\beta}_{2}^{'} \right)+\left( \hat{\beta}_{2}^{'} \right)^{2}Var\left( \hat{\alpha}_{2}^{'} \right)$ | (8) |
| --- | --- | --- |
|  | $Var\left( \hat{\delta}_{2}^{'} \right)=Var\left( \hat{\tau}^{'}-\hat{\alpha}_{2}^{'}\hat{\beta}_{2}^{'} \right)=Var\left( \hat{\tau}^{'} \right)+Var\left( \hat{\alpha}_{2}^{'}\hat{\beta}_{2}^{'} \right)$ | (9) |
|  | $Var\left( \frac{\hat{\alpha}_{2}^{'}\hat{\beta}_{2}^{'}}{\hat{\tau}^{'}} \right)\approx\frac{Var\left( \hat{\alpha}_{2}^{'}\hat{\beta}_{2}^{'} \right)}{{(\hat{\tau}^{'})}^{2}}+\frac{\left( \hat{\alpha}_{2}^{'}\hat{\beta}_{2}^{'} \right)^{2}Var\left( \hat{\tau}^{'} \right)}{{(\hat{\tau}^{'})}^{4}}.$ | (10) |

This completes the statistical inference for the MR-based product method.

# Appendix 3. Additional Simulation

In this section, we performed an additional simulation for binary outcomes using the same parameter settings and data generating process of the simulation for continuous outcome. The only difference is that the binary outcome, denoted as $Y_{b}$, is generated based on a logistic regression model using the probability equation $P(Y_{b}=1)=\frac{1}{1+exp\left( -\left( c+Y \right) \right)}$, where $Y$ follows Equation (12) of the main paper, and the constant $c$ is chosen such that the prevalence of $Y_{b}=1$ is approximately 3% in each scenario. We adopted the case-control sampling scheme and generated 30,000 cases and 50,000 controls to produce the summary data of $Y$ in the simulation. Recall we set ($\alpha, \beta, \delta$)=(0.3,0.3,0.21) with the mediation proportion 0.3, we expect that the of MR-based mediation estimates would provide good approximations for these parameters under the rare-outcome assumption (typically prevalence <10%).[^6-8^](#_ENREF_6)

Fig. S1 presents the comparisons of the estimators for TE, DE, IE across different approaches based on MSEs. Similar to the main findings in the simulation for continuous outcomes, the IVW-based methods performed well in the scenarios with null or balanced pleiotropy (S1 – S6) while the median-based methods were more robust to the directional pleiotropy. In Fig. S2, we found the coverage rates may be slightly dropped due to the non-collapsibility of odds ratio. For example, in S1 where no pleiotropy is imposed, the coverage rate for DE based-on Diff-IVW dropped from 0.92 to 0.87 comparing to the continuous outcome simulation. In conclusion, the overall pattern in the simulation for binary outcomes are similar to the simulation for the continuous outcomes which we have discussed in the main paper. The corresponding numerical results are provided in Supplementary Appendix 5.

| 1. TE |
| --- |
| 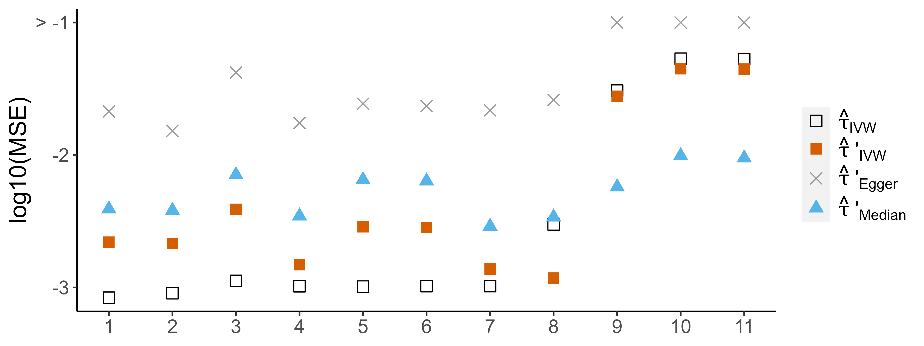 |
| 1. DE |
| 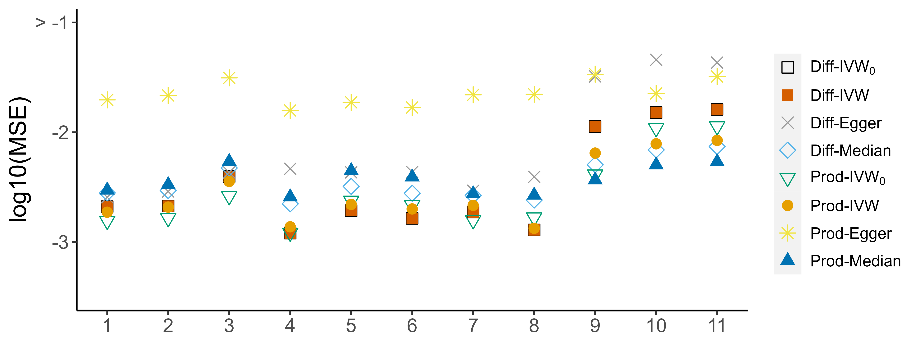 |
| 1. IE |
| 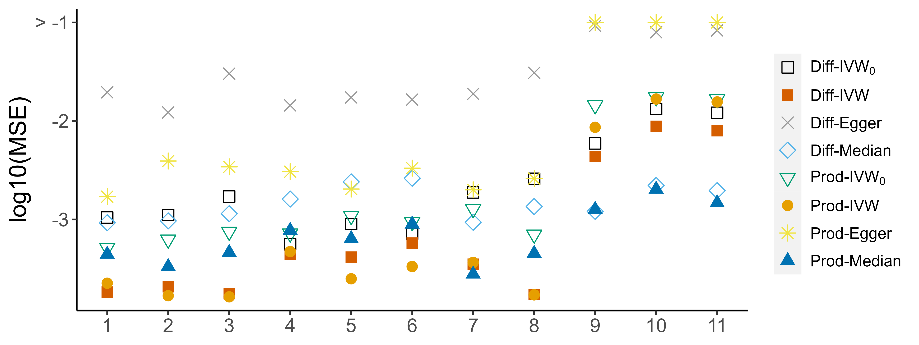 |
| 1. $\rho$ |
| 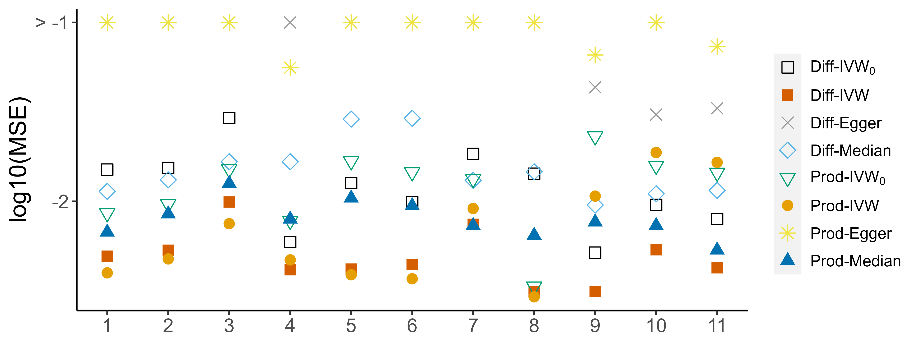 |

**Fig. S1** Logarithm of the mean squared errors (MSEs) for (a) total effect (TE), (b)direct effect (DE), (c) indirect effect (IE), and (d) mediation proportion ($\rho$). Lower points on the graph indicate better accuracy.

| (a) DE |
| --- |
| 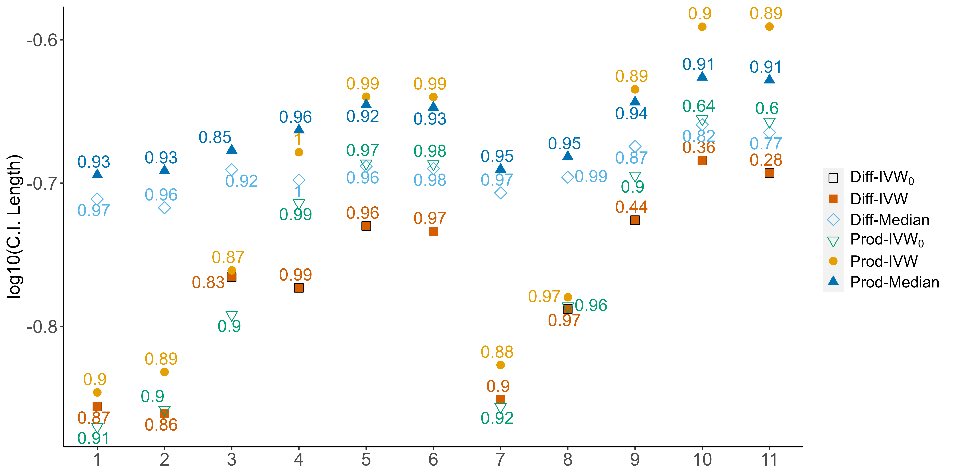 |
| (b) IE |
| 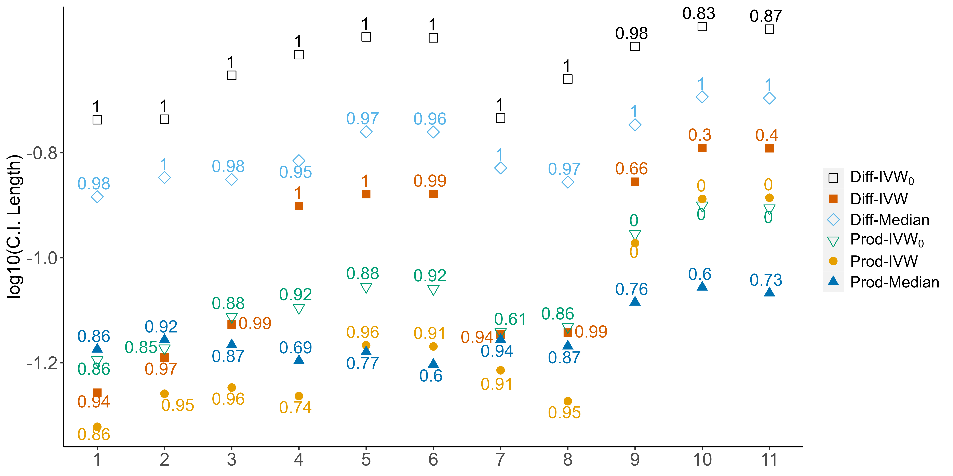 |
| (c) $\rho$ |
| 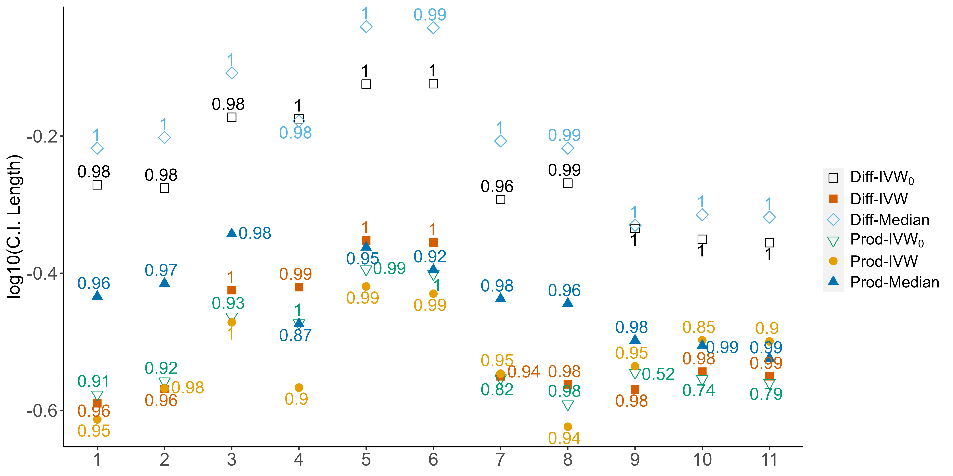 |

**Fig. S2** Logarithm of the interval lengths and empirical coverage rates (shown in number) of the 95% confidence intervals for (a) direct effect (DE), (b) indirect effect (IE), and (c) mediation proportion ($\rho$)

# Appendix 4. Supplements for the applications

The genetic association estimates were obtained from several public GWAS summary data: the GIANT-UK Biobank GWAS meta-analysis for WHR (697,734 individuals of European ancestry)[^9^](#_ENREF_9), the CARDIoGRAMplusC4D consortium GWAS for CAD (60, 801 cases and 123,504 controls primarily of European ancestry (77%)),[^10^](#_ENREF_10) the DIAGRAM Consortium GWAS meta-analysis for T2D (74,124 cases and 824,006 controls of European ancestry)[^11^](#_ENREF_11) and the lifetime smoking measure (SMK) GWAS from UK Biobank (462,690 individuals of European ancestry).[^12^](#_ENREF_12) To select the genetic instruments $G_{X}$ for WHR, the genome-wide significant SNPs (association *P*$<5\times{10}^{-8}$) were clumped for independence using the R package: TwoSampleMR[^13^](#_ENREF_13) with the default settings ($r^{2}$ threshold = 0.001 and window size 10,000 kb). The same principles were applied to obtain $G_{M}$ for the potential mediators of SMK and T2D. Then, we performed double clumping on $G_{X} \cup G_{M}$using the same clumping criteria to obtain the genetic instruments $G_{X}^{'}$ and $G_{M}^{'}$. Detailed information for the selected SNPs is provided in Supplementary Table (XLS).

In our study, the GWAS we used for WHR and T2D shared overlapping samples from UK Biobank (485,486 out of 697,734 for WHR and 442,817 out of 898,130 for T2D). In the main paper, we conclude that T2D significantly mediates the effect of WHR on CAD based on the three recommended methods (Diff-IVW, Prod-IVW, Prod-Median). However, since Prod-IVW and Prod-Median assume a three-sample setting, the overlapping samples from UK Biobank between WHR and T2D raise concerns for those methods. To strengthen our findings, we performed additional analyses in the three-sample setting by utilizing an earlier GWAS of T2D from DIAGRAM Consortium[^14^](#_ENREF_14) (26,676 cases and

132,532 controls) which do not include the UK Biobank participants. The results (presented in Table S1) are mainly consistent with Table 4 in the main paper, and all the three recommended methods (Diff-IVW, Prod-IVW, Prod-Median) indicate that T2D significantly mediates the effect of WHR on CAD at a 95% confidence level.

At the end of the section, we provide the average $F$-statistic and the two-sample conditional $F$-statistics[^15^](#_ENREF_15) in Table S2 to assess the strength of the genetic instruments $G_{X}^{'}$ and $G_{M}^{'}$.

**Table S1** MR-based mediation analysis for WHR (exposure), T2D (mediator, no UK Biobank participants), and CAD (outcome). The odds ratios of TE, DE and IE and the mediation proportion are provided with 95% confidence intervals

| Method | TE | DE | IE | $\rho$ |
| --- | --- | --- | --- | --- |
| Diff-IVW_0_ | 1.57(1.41,1.75) | 1.44(1.26,1.64) | 1.09(0.92,1.29) | 0.198(-0.146,0.541) |
| Diff-IVW | 1.59(1.41,1.79) | 1.44(1.26,1.64) | 1.11(1.05,1.16) | 0.217(0.091,0.343) |
| Diff-Egger | 1.33(0.95,1.87) | 1.26(0.93,1.72) | 1.05(0.9,1.23) | 0.179(-0.314,0.672) |
| Diff-Median | 1.51(1.33,1.71) | 1.43(1.29,1.67) | 1.06(0.93,1.14) | 0.134(-0.208,0.302) |
| Prod-IVW_0_ | 1.57(1.41,1.75) | 1.43(1.27,1.61) | 1.1(1.04,1.16) | 0.212(0.086,0.338) |
| Prod-IVW | 1.59(1.41,1.79) | 1.43(1.24,1.64) | 1.12(1.03,1.21) | 0.235(0.054,0.415) |
| Prod-Egger | 1.33(0.95,1.87) | 1.25(0.87,1.78) | 1.07(0.96,1.19) | 0.225(-0.24,0.69) |
| Prod-Median | 1.51(1.32,1.72) | 1.37(1.19,1.59) | 1.1(1.04,1.16) | 0.229(0.079,0.379) |

TE=total effect, DE=direct effect, IE=indirect effect, and $\rho$ is the mediation proportion.

**Table S2** Average $F$-statistic and two-sample conditional $F$-statistic of the genetic instruments $G_{X}^{'}$ and $G_{M}^{'}$ for the three MR-based mediation analysis

| Cases for  Mediation Analysis | Genetic Instruments | Average  $F$-statistic | Two-sample conditional  $F$-statistic |
| --- | --- | --- | --- |
| Exposure: WHR  Mediator: SMK  Outcome: CAD | $G_{X}^{'}$ | 67.6 | 35.4 |
|  | $G_{M}^{'}$ | 51.5 | 9.7 |
| Exposure: WHR  Mediator: T2D  Outcome: CAD | $G_{X}^{'}$ | 69.6 | 37.0 |
|  | $G_{M}^{'}$ | 91.5 | 26.5 |
| Exposure: WHR  Mediator: T2D (no UKB)  Outcome: CAD | $G_{X}^{'}$ | 64.5 | 17.9 |
|  | $G_{M}^{'}$ | 91.1 | 6.9 |

# Appendix 5. Numerical results for the simulations

Table S3 to Table S24 provide the numerical results for the eleven simulation scenarios as discussed in the main paper. For example, Table S3 reports the mean squared errors (MSEs) and Table S4 presents the empirical coverage rates and interval lengths of the 95% CI for Scenario 1. Table S5 and Table S6 provide the corresponding results for Scenario 2, and so on.

Similarly, Table S25 to Table S46 provide the numerical results corresponding to the eleven scenarios for the additional simulation conducted for binary outcomes, as described in Supplementary Appendix 3.

| **Table S3** Mean and mean squared error (MSE) of the estimate for Scenario 1$(\alpha=0.3, \beta=0.3, \delta=0.21,\tau=0.3, \rho=0.3)$ | | | | | | | | | |  |
| --- | --- | --- | --- | --- | --- | --- | --- | --- | --- | --- |
| Method | | Total effect  $(\tau=0.3)$ | | Direct effect  $(\delta=0.21)$ | | Indirect effect  $(\alpha\beta=0.09)$ | | Mediation proportion  $(\rho=0.3)$ | |  |
|  |  | Mean | MSE | Mean | MSE | Mean | MSE | Mean | MSE |  |
| Difference | Diff-IVW_0_ | 0.32640 | 0.00114 | 0.19965 | 0.00074 | 0.12675 | 0.00157 | 0.38980 | 0.01064 |  |
|  | Diff-IVW | 0.29380 | 0.00059 | 0.19965 | 0.00074 | 0.09415 | 0.00011 | 0.32241 | 0.00222 |  |
|  | Diff-Egger | 0.22614 | 0.01105 | 0.23016 | 0.00174 | -0.00402 | 0.01354 | 0.30983 | 162.26987 |  |
|  | Diff-Median | 0.27946 | 0.00127 | 0.20054 | 0.00113 | 0.07892 | 0.00050 | 0.28431 | 0.00546 |  |
| Product | Prod-IVW_0_ | 0.32640 | 0.00114 | 0.20880 | 0.00056 | 0.11760 | 0.00088 | 0.36176 | 0.00550 |  |
|  | Prod-IVW | 0.29380 | 0.00059 | 0.20470 | 0.00067 | 0.08910 | 0.00008 | 0.30527 | 0.00161 |  |
|  | Prod-Egger | 0.22614 | 0.01105 | 0.14660 | 0.01074 | 0.07954 | 0.00107 | 0.18002 | 44.68114 |  |
|  | Prod-Median | 0.27946 | 0.00127 | 0.19692 | 0.00118 | 0.08254 | 0.00018 | 0.29897 | 0.00284 |  |
| **Table S4** Coverage rate (C.R.) and interval length (I.L.) of the 95% confidence interval for Scenario 1 | | | | | | | | | |  |
| Method | | Total effect  $(\tau=0.3)$ | | Direct effect  $(\delta=0.21)$ | | Indirect effect  $(\alpha\beta=0.09)$ | | Mediation proportion  $(\rho=0.3)$ | |  |
|  |  | C.R. | I.L. | C.R. | I.L. | C.R. | I.L. | C.R. | I.L. |  |
| Difference | Diff-IVW_0_ | 0.777 | 0.08487 | 0.923 | 0.09782 | 0.982 | 0.12957 | 0.948 | 0.34031 |  |
|  | Diff-IVW | 0.943 | 0.09216 | 0.923 | 0.09782 | 0.941 | 0.04102 | 0.944 | 0.16723 |  |
|  | Diff-Egger | 0.852 | 0.31161 | 0.919 | 0.14356 | 0.739 | 0.28406 | 1 | 306.40724 |  |
|  | Diff-Median | 0.876 | 0.12964 | 0.99 | 0.13608 | 0.987 | 0.10295 | 0.994 | 0.38964 |  |
| Product | Prod-IVW_0_ | 0.777 | 0.08487 | 0.957 | 0.09954 | 0.43 | 0.05189 | 0.814 | 0.18578 |  |
|  | Prod-IVW | 0.943 | 0.09216 | 0.94 | 0.09966 | 0.955 | 0.03782 | 0.965 | 0.16205 |  |
|  | Prod-Egger | 0.852 | 0.31161 | 0.899 | 0.33486 | 0.907 | 0.12065 | 0.961 | 160.87279 |  |
|  | Prod-Median | 0.938 | 0.13139 | 0.961 | 0.14174 | 0.933 | 0.05302 | 0.966 | 0.24039 |  |
| **Table S5** Mean and mean squared error (MSE) of the estimate for Scenario 2$(\alpha=0.3, \beta=0.3, \delta=0.21,\tau=0.3, \rho=0.3)$ | | | | | | | | | | |
| Method | | Total effect  $(\tau=0.3)$ | | Direct effect  $(\delta=0.21)$ | | Indirect effect  $(\alpha\beta=0.09)$ | | Mediation proportion  $(\rho=0.3)$ | | |
|  |  | Mean | MSE | Mean | MSE | Mean | MSE | Mean | MSE | |
| Difference | Diff-IVW_0_ | 0.32761 | 0.00121 | 0.20037 | 0.00071 | 0.12723 | 0.00162 | 0.38965 | 0.01064 | |
|  | Diff-IVW | 0.29604 | 0.00057 | 0.20037 | 0.00071 | 0.09567 | 0.00015 | 0.32485 | 0.00247 | |
|  | Diff-Egger | 0.26858 | 0.00680 | 0.22615 | 0.00171 | 0.04243 | 0.00711 | 0.08304 | 0.21951 | |
|  | Diff-Median | 0.28314 | 0.00114 | 0.20118 | 0.00111 | 0.08196 | 0.00056 | 0.29057 | 0.00634 | |
| Product | Prod-IVW_0_ | 0.32761 | 0.00121 | 0.20842 | 0.00057 | 0.11919 | 0.00097 | 0.36532 | 0.00597 | |
|  | Prod-IVW | 0.29604 | 0.00057 | 0.20272 | 0.00070 | 0.09332 | 0.00009 | 0.31730 | 0.00196 | |
|  | Prod-Egger | 0.26858 | 0.00680 | 0.13383 | 0.01328 | 0.13475 | 0.00380 | 0.55876 | 0.17711 | |
|  | Prod-Median | 0.28314 | 0.00114 | 0.19631 | 0.00121 | 0.08683 | 0.00014 | 0.31028 | 0.00299 | |
| **Table S6** Coverage rate (C.R.) and interval length (I.L.) of the 95% confidence interval for Scenario 2 | | | | | | | | | |  |
| Method | | Total effect  $(\tau=0.3)$ | | Direct effect  $(\delta=0.21)$ | | Indirect effect  $(\alpha\beta=0.09)$ | | Mediation proportion  $(\rho=0.3)$ | |  |
|  |  | C.R. | I.L. | C.R. | I.L. | C.R. | I.L. | C.R. | I.L. |  |
| Difference | Diff-IVW_0_ | 0.782 | 0.08808 | 0.924 | 0.09640 | 0.972 | 0.13067 | 0.945 | 0.33819 |  |
|  | Diff-IVW | 0.952 | 0.09545 | 0.924 | 0.09640 | 0.934 | 0.04726 | 0.936 | 0.17185 |  |
|  | Diff-Egger | 0.949 | 0.32413 | 0.929 | 0.14841 | 0.929 | 0.29533 | 0.99 | 1.40639 |  |
|  | Diff-Median | 0.912 | 0.13117 | 0.986 | 0.13420 | 0.991 | 0.11034 | 0.996 | 0.40416 |  |
| Product | Prod-IVW_0_ | 0.782 | 0.08808 | 0.962 | 0.10456 | 0.456 | 0.05620 | 0.846 | 0.19885 |  |
|  | Prod-IVW | 0.952 | 0.09545 | 0.95 | 0.10660 | 0.991 | 0.04725 | 0.992 | 0.19141 |  |
|  | Prod-Egger | 0.949 | 0.32413 | 0.901 | 0.37163 | 0.916 | 0.17958 | 0.995 | 1.25383 |  |
|  | Prod-Median | 0.954 | 0.13271 | 0.957 | 0.14392 | 0.971 | 0.05555 | 0.98 | 0.24848 |  |
| **Table S7** Mean and mean squared error (MSE) of the estimate for Scenario 3$(\alpha=0.3, \beta=0.3, \delta=0.21,\tau=0.3, \rho=0.3)$ | | | | | | | | | |  |
| Method | | Total effect  $(\tau=0.3)$ | | Direct effect  $(\delta=0.21)$ | | Indirect effect  $(\alpha\beta=0.09)$ | | Mediation proportion  $(\rho=0.3)$ | |  |
|  |  | Mean | MSE | Mean | MSE | Mean | MSE | Mean | MSE |  |
| Difference | Diff-IVW_0_ | 0.31226 | 0.00061 | 0.17263 | 0.00206 | 0.13963 | 0.00270 | 0.44914 | 0.02553 |  |
|  | Diff-IVW | 0.27186 | 0.00137 | 0.17263 | 0.00206 | 0.09923 | 0.00020 | 0.36775 | 0.00716 |  |
|  | Diff-Egger | 0.15256 | 0.03015 | 0.19523 | 0.00235 | -0.04267 | 0.02471 | -0.56282 | 62.37143 |  |
|  | Diff-Median | 0.25341 | 0.00323 | 0.17786 | 0.00225 | 0.07555 | 0.00073 | 0.30032 | 0.00846 |  |
| Product | Prod-IVW_0_ | 0.31226 | 0.00061 | 0.18721 | 0.00109 | 0.12505 | 0.00136 | 0.40227 | 0.01252 |  |
|  | Prod-IVW | 0.27186 | 0.00137 | 0.17726 | 0.00173 | 0.09461 | 0.00011 | 0.35077 | 0.00477 |  |
|  | Prod-Egger | 0.15256 | 0.03015 | 0.09625 | 0.02240 | 0.05631 | 0.00218 | 0.52064 | 7.57789 |  |
|  | Prod-Median | 0.25341 | 0.00323 | 0.17147 | 0.00270 | 0.08194 | 0.00019 | 0.32911 | 0.00495 |  |
| **Table S8** Coverage rate (C.R.) and interval length (I.L.) of the 95% confidence interval for Scenario 3 | | | | | | | | | |  |
| Method | | Total effect  $(\tau=0.3)$ | | Direct effect  $(\delta=0.21)$ | | Indirect effect  $(\alpha\beta=0.09)$ | | Mediation proportion  $(\rho=0.3)$ | |  |
|  |  | C.R. | I.L. | C.R. | I.L. | C.R. | I.L. | C.R. | I.L. |  |
| Difference | Diff-IVW_0_ | 0.983 | 0.12248 | 0.927 | 0.14912 | 0.999 | 0.19304 | 0.981 | 0.52657 |  |
|  | Diff-IVW | 0.96 | 0.14222 | 0.927 | 0.14912 | 0.98 | 0.06405 | 0.983 | 0.29025 |  |
|  | Diff-Egger | 0.84 | 0.48067 | 0.973 | 0.21957 | 0.841 | 0.43858 | 0.999 | 179.24894 |  |
|  | Diff-Median | 0.529 | 0.14110 | 0.904 | 0.14760 | 0.983 | 0.11526 | 1 | 0.50293 |  |
| Product | Prod-IVW_0_ | 0.983 | 0.12248 | 0.972 | 0.14145 | 0.512 | 0.07063 | 0.854 | 0.27780 |  |
|  | Prod-IVW | 0.96 | 0.14222 | 0.95 | 0.15125 | 0.993 | 0.05122 | 0.995 | 0.26680 |  |
|  | Prod-Egger | 0.84 | 0.48067 | 0.928 | 0.50773 | 0.871 | 0.16003 | 0.977 | 52.84747 |  |
|  | Prod-Median | 0.776 | 0.14170 | 0.86 | 0.15175 | 0.935 | 0.05416 | 0.986 | 0.29001 |  |
| **Table S9** Mean and mean squared error (MSE) of the estimate for Scenario 4$(\alpha=0.3, \beta=0.3, \delta=0.21,\tau=0.3, \rho=0.3)$ | | | | | | | | | |  |
| Method | | Total effect  $(\tau=0.3)$ | | Direct effect  $(\delta=0.21)$ | | Indirect effect  $(\alpha\beta=0.09)$ | | Mediation proportion  $(\rho=0.3)$ | |  |
|  |  | Mean | MSE | Mean | MSE | Mean | MSE | Mean | MSE |  |
| Difference | Diff-IVW_0_ | 0.34140 | 0.00222 | 0.22987 | 0.00105 | 0.11153 | 0.00070 | 0.32774 | 0.00301 |  |
|  | Diff-IVW | 0.31161 | 0.00077 | 0.22987 | 0.00105 | 0.08175 | 0.00018 | 0.26355 | 0.00270 |  |
|  | Diff-Egger | 0.30484 | 0.01329 | 0.26412 | 0.00479 | 0.04072 | 0.01242 | 0.01217 | 0.64025 |  |
|  | Diff-Median | 0.28588 | 0.00134 | 0.21679 | 0.00125 | 0.06910 | 0.00102 | 0.24196 | 0.01016 |  |
| Product | Prod-IVW_0_ | 0.34140 | 0.00222 | 0.21680 | 0.00063 | 0.12460 | 0.00131 | 0.36645 | 0.00590 |  |
|  | Prod-IVW | 0.31161 | 0.00077 | 0.23358 | 0.00125 | 0.07804 | 0.00021 | 0.25207 | 0.00343 |  |
|  | Prod-Egger | 0.30484 | 0.01329 | 0.25916 | 0.01309 | 0.04569 | 0.00283 | 0.15923 | 0.03269 |  |
|  | Prod-Median | 0.28588 | 0.00134 | 0.21319 | 0.00132 | 0.07269 | 0.00042 | 0.25825 | 0.00448 |  |
| **Table S10** Coverage rate (C.R.) and interval length (I.L.) of the 95% confidence interval for Scenario 4 | | | | | | | | | |  |
| Method | | Total effect  $(\tau=0.3)$ | | Direct effect  $(\delta=0.21)$ | | Indirect effect  $(\alpha\beta=0.09)$ | | Mediation proportion  $(\rho=0.3)$ | |  |
|  |  | C.R. | I.L. | C.R. | I.L. | C.R. | I.L. | C.R. | I.L. |  |
| Difference | Diff-IVW_0_ | 0.957 | 0.16497 | 0.98 | 0.14610 | 1 | 0.22041 | 1 | 0.53958 |  |
|  | Diff-IVW | 0.999 | 0.19068 | 0.98 | 0.14610 | 1 | 0.13227 | 1 | 0.34637 |  |
|  | Diff-Egger | 0.945 | 0.53211 | 0.901 | 0.22322 | 0.976 | 0.48499 | 0.975 | 3.16648 |  |
|  | Diff-Median | 0.956 | 0.14604 | 0.995 | 0.14633 | 0.969 | 0.12142 | 0.981 | 0.44621 |  |
| Product | Prod-IVW_0_ | 0.957 | 0.16497 | 1 | 0.18229 | 0.674 | 0.07744 | 0.998 | 0.28967 |  |
|  | Prod-IVW | 0.999 | 0.19068 | 0.997 | 0.19755 | 0.913 | 0.05139 | 0.918 | 0.22836 |  |
|  | Prod-Egger | 0.945 | 0.53211 | 0.96 | 0.54555 | 0.581 | 0.11616 | 0.672 | 0.62742 |  |
|  | Prod-Median | 0.953 | 0.14711 | 0.97 | 0.15614 | 0.742 | 0.05213 | 0.849 | 0.23088 |  |
| **Table S11** Mean and mean squared error (MSE) of the estimate for Scenario 5$(\alpha=0.3, \beta=0.3, \delta=0.21,\tau=0.3, \rho=0.3)$ | | | | | | | | | |  |
| Method | | Total effect  $(\tau=0.3)$ | | Direct effect  $(\delta=0.21)$ | | Indirect effect  $(\alpha\beta=0.09)$ | | Mediation proportion  $(\rho=0.3)$ | |  |
|  |  | Mean | MSE | Mean | MSE | Mean | MSE | Mean | MSE |  |
| Difference | Diff-IVW_0_ | 0.32558 | 0.00117 | 0.20238 | 0.00072 | 0.12320 | 0.00136 | 0.37971 | 0.00911 |  |
|  | Diff-IVW | 0.28723 | 0.00081 | 0.20238 | 0.00072 | 0.08485 | 0.00018 | 0.29688 | 0.00207 |  |
|  | Diff-Egger | 0.24688 | 0.01690 | 0.21579 | 0.00292 | 0.03109 | 0.01392 | -0.10930 | 2.05344 |  |
|  | Diff-Median | 0.25821 | 0.00312 | 0.19424 | 0.00156 | 0.06398 | 0.00160 | 0.24519 | 0.01613 |  |
| Product | Prod-IVW_0_ | 0.32558 | 0.00117 | 0.19447 | 0.00085 | 0.13111 | 0.00181 | 0.40458 | 0.01279 |  |
|  | Prod-IVW | 0.28723 | 0.00081 | 0.20114 | 0.00080 | 0.08610 | 0.00009 | 0.30211 | 0.00167 |  |
|  | Prod-Egger | 0.24688 | 0.01690 | 0.18286 | 0.01279 | 0.06402 | 0.00173 | 0.33777 | 0.34630 |  |
|  | Prod-Median | 0.25821 | 0.00312 | 0.18417 | 0.00220 | 0.07404 | 0.00039 | 0.29323 | 0.00433 |  |
| **Table S12** Coverage rate (C.R.) and interval length (I.L.) of the 95% confidence interval for Scenario 5 | | | | | | | | | |  |
| Method | | Total effect  $(\tau=0.3)$ | | Direct effect  $(\delta=0.21)$ | | Indirect effect  $(\alpha\beta=0.09)$ | | Mediation proportion  $(\rho=0.3)$ | |  |
|  |  | C.R. | I.L. | C.R. | I.L. | C.R. | I.L. | C.R. | I.L. |  |
| Difference | Diff-IVW_0_ | 0.997 | 0.17822 | 1 | 0.17133 | 1 | 0.24727 | 1 | 0.62941 |  |
|  | Diff-IVW | 1 | 0.21127 | 1 | 0.17133 | 1 | 0.13811 | 1 | 0.39886 |  |
|  | Diff-Egger | 0.981 | 0.58969 | 0.988 | 0.27374 | 0.98 | 0.52643 | 0.974 | 11.22414 |  |
|  | Diff-Median | 0.711 | 0.15883 | 0.978 | 0.15314 | 0.951 | 0.14252 | 0.993 | 0.61243 |  |
| Product | Prod-IVW_0_ | 0.997 | 0.17822 | 0.999 | 0.19834 | 0.604 | 0.08691 | 0.995 | 0.34958 |  |
|  | Prod-IVW | 1 | 0.21127 | 1 | 0.22199 | 0.998 | 0.06790 | 0.997 | 0.32888 |  |
|  | Prod-Egger | 0.981 | 0.58969 | 0.991 | 0.61089 | 0.854 | 0.15472 | 0.957 | 4.59939 |  |
|  | Prod-Median | 0.832 | 0.15797 | 0.927 | 0.16724 | 0.782 | 0.05469 | 0.947 | 0.28781 |  |
| **Table S13** Mean and mean squared error (MSE) of the estimate for Scenario 6$(\alpha=0.3, \beta=0.3, \delta=0.21,\tau=0.3, \rho=0.3)$ | | | | | | | | | |  |
| Method | | Total effect  $(\tau=0.3)$ | | Direct effect  $(\delta=0.21)$ | | Indirect effect  $(\alpha\beta=0.09)$ | | Mediation proportion  $(\rho=0.3)$ | |  |
|  |  | Mean | MSE | Mean | MSE | Mean | MSE | Mean | MSE |  |
| Difference | Diff-IVW_0_ | 0.32576 | 0.00120 | 0.20876 | 0.00063 | 0.11700 | 0.00098 | 0.36021 | 0.00617 |  |
|  | Diff-IVW | 0.28762 | 0.00083 | 0.20876 | 0.00063 | 0.07886 | 0.00028 | 0.27521 | 0.00248 |  |
|  | Diff-Egger | 0.25456 | 0.01736 | 0.21268 | 0.00293 | 0.04188 | 0.01395 | 0.37498 | 126.91324 |  |
|  | Diff-Median | 0.25836 | 0.00308 | 0.20078 | 0.00139 | 0.05759 | 0.00192 | 0.22006 | 0.01888 |  |
| Product | Prod-IVW_0_ | 0.32576 | 0.00120 | 0.19760 | 0.00076 | 0.12816 | 0.00158 | 0.39515 | 0.01083 |  |
|  | Prod-IVW | 0.28762 | 0.00083 | 0.20501 | 0.00074 | 0.08261 | 0.00014 | 0.28933 | 0.00171 |  |
|  | Prod-Egger | 0.25456 | 0.01736 | 0.21015 | 0.01262 | 0.04442 | 0.00292 | 0.14660 | 2.11238 |  |
|  | Prod-Median | 0.25836 | 0.00308 | 0.19046 | 0.00190 | 0.06791 | 0.00064 | 0.26873 | 0.00529 |  |
| **Table S14** Coverage rate (C.R.) and interval length (I.L.) of the 95% confidence interval for Scenario 6 | | | | | | | | | |  |
| Method | | Total effect  $(\tau=0.3)$ | | Direct effect  $(\delta=0.21)$ | | Indirect effect  $(\alpha\beta=0.09)$ | | Mediation proportion  $(\rho=0.3)$ | |  |
|  |  | C.R. | I.L. | C.R. | I.L. | C.R. | I.L. | C.R. | I.L. |  |
| Difference | Diff-IVW_0_ | 0.996 | 0.17791 | 0.999 | 0.16924 | 1 | 0.24561 | 1 | 0.62920 |  |
|  | Diff-IVW | 1 | 0.21095 | 0.999 | 0.16924 | 1 | 0.13829 | 1 | 0.39896 |  |
|  | Diff-Egger | 0.974 | 0.58831 | 0.99 | 0.27178 | 0.973 | 0.52461 | 0.965 | 372.56605 |  |
|  | Diff-Median | 0.725 | 0.15883 | 0.995 | 0.15191 | 0.902 | 0.14212 | 0.976 | 0.61182 |  |
| Product | Prod-IVW_0_ | 0.996 | 0.17791 | 1 | 0.19780 | 0.705 | 0.08631 | 0.999 | 0.34442 |  |
|  | Prod-IVW | 1 | 0.21095 | 1 | 0.22169 | 0.994 | 0.06790 | 0.995 | 0.32176 |  |
|  | Prod-Egger | 0.974 | 0.58831 | 0.982 | 0.60626 | 0.68 | 0.14103 | 0.851 | 47.96735 |  |
|  | Prod-Median | 0.84 | 0.15792 | 0.952 | 0.16654 | 0.618 | 0.05267 | 0.871 | 0.27150 |  |
| **Table S15** Mean and mean squared error (MSE) of the estimate for Scenario 7$(\alpha=0.3, \beta=0.3, \delta=0.21,\tau=0.3, \rho=0.3)$ | | | | | | | | | |  |
| Method | | Total effect  $(\tau=0.3)$ | | Direct effect  $(\delta=0.21)$ | | Indirect effect  $(\alpha\beta=0.09)$ | | Mediation proportion  $(\rho=0.3)$ | |  |
|  |  | Mean | MSE | Mean | MSE | Mean | MSE | Mean | MSE |  |
| Difference | Diff-IVW_0_ | 0.34286 | 0.00228 | 0.20168 | 0.00071 | 0.14119 | 0.00289 | 0.41305 | 0.01550 |  |
|  | Diff-IVW | 0.31104 | 0.00068 | 0.20168 | 0.00071 | 0.10936 | 0.00053 | 0.35331 | 0.00490 |  |
|  | Diff-Egger | 0.22689 | 0.01100 | 0.22551 | 0.00167 | 0.00138 | 0.01256 | -0.08347 | 1.11422 |  |
|  | Diff-Median | 0.29168 | 0.00096 | 0.20288 | 0.00110 | 0.08881 | 0.00052 | 0.30555 | 0.00601 |  |
| Product | Prod-IVW_0_ | 0.34286 | 0.00228 | 0.20866 | 0.00060 | 0.13420 | 0.00210 | 0.39294 | 0.01056 |  |
|  | Prod-IVW | 0.31104 | 0.00068 | 0.19680 | 0.00084 | 0.11424 | 0.00069 | 0.36945 | 0.00675 |  |
|  | Prod-Egger | 0.22689 | 0.01100 | 0.13854 | 0.01191 | 0.08835 | 0.00122 | 0.43436 | 0.26751 |  |
|  | Prod-Median | 0.29168 | 0.00096 | 0.20032 | 0.00117 | 0.09137 | 0.00014 | 0.31692 | 0.00325 |  |
| **Table S16** Coverage rate (C.R.) and interval length (I.L.) of the 95% confidence interval for Scenario 7 | | | | | | | | | |  |
| Method | | Total effect  $(\tau=0.3)$ | | Direct effect  $(\delta=0.21)$ | | Indirect effect  $(\alpha\beta=0.09)$ | | Mediation proportion  $(\rho=0.3)$ | |  |
|  |  | C.R. | I.L. | C.R. | I.L. | C.R. | I.L. | C.R. | I.L. |  |
| Difference | Diff-IVW_0_ | 0.524 | 0.08705 | 0.937 | 0.09908 | 0.823 | 0.13197 | 0.836 | 0.32624 |  |
|  | Diff-IVW | 0.937 | 0.09601 | 0.937 | 0.09908 | 0.707 | 0.05315 | 0.841 | 0.18332 |  |
|  | Diff-Egger | 0.883 | 0.32357 | 0.937 | 0.15022 | 0.776 | 0.29329 | 1 | 3.32612 |  |
|  | Diff-Median | 0.963 | 0.13160 | 0.991 | 0.13785 | 0.998 | 0.11451 | 0.999 | 0.40398 |  |
| Product | Prod-IVW_0_ | 0.524 | 0.08705 | 0.961 | 0.10541 | 0.086 | 0.05931 | 0.6 | 0.20076 |  |
|  | Prod-IVW | 0.937 | 0.09601 | 0.937 | 0.10949 | 0.584 | 0.05241 | 0.849 | 0.20527 |  |
|  | Prod-Egger | 0.883 | 0.32357 | 0.905 | 0.35865 | 0.944 | 0.15170 | 0.981 | 1.86083 |  |
|  | Prod-Median | 0.972 | 0.13346 | 0.966 | 0.14516 | 0.987 | 0.05695 | 0.983 | 0.24742 |  |
| **Table S17** Mean and mean squared error (MSE) of the estimate for Scenario 8$(\alpha=0.3, \beta=0.3, \delta=0.21,\tau=0.3, \rho=0.3)$ | | | | | | | | | |  |
| Method | | Total effect  $(\tau=0.3)$ | | Direct effect  $(\delta=0.21)$ | | Indirect effect  $(\alpha\beta=0.09)$ | | Mediation proportion  $(\rho=0.3)$ | |  |
|  |  | Mean | MSE | Mean | MSE | Mean | MSE | Mean | MSE |  |
| Difference | Diff-IVW_0_ | 0.37961 | 0.00681 | 0.23179 | 0.00115 | 0.14782 | 0.00358 | 0.39056 | 0.01025 |  |
|  | Diff-IVW | 0.33032 | 0.00150 | 0.23179 | 0.00115 | 0.09852 | 0.00018 | 0.29979 | 0.00145 |  |
|  | Diff-Egger | 0.20398 | 0.01718 | 0.26085 | 0.00440 | -0.05687 | 0.02826 | -0.64436 | 43.29621 |  |
|  | Diff-Median | 0.28013 | 0.00138 | 0.21084 | 0.00118 | 0.06929 | 0.00089 | 0.24884 | 0.00879 |  |
| Product | Prod-IVW_0_ | 0.37961 | 0.00681 | 0.25727 | 0.00282 | 0.12234 | 0.00118 | 0.32328 | 0.00176 |  |
|  | Prod-IVW | 0.33032 | 0.00150 | 0.23757 | 0.00143 | 0.09275 | 0.00009 | 0.28232 | 0.00153 |  |
|  | Prod-Egger | 0.20398 | 0.01718 | 0.13857 | 0.01400 | 0.06541 | 0.00159 | 0.45429 | 3.64836 |  |
|  | Prod-Median | 0.28013 | 0.00138 | 0.19876 | 0.00128 | 0.08137 | 0.00020 | 0.29458 | 0.00303 |  |
| **Table S18** Coverage rate (C.R.) and interval length (I.L.) of the 95% confidence interval for Scenario 8 | | | | | | | | | |  |
| Method | | Total effect  $(\tau=0.3)$ | | Direct effect  $(\delta=0.21)$ | | Indirect effect  $(\alpha\beta=0.09)$ | | Mediation proportion  $(\rho=0.3)$ | |  |
|  |  | C.R. | I.L. | C.R. | I.L. | C.R. | I.L. | C.R. | I.L. |  |
| Difference | Diff-IVW_0_ | 0.229 | 0.12761 | 0.957 | 0.13592 | 0.994 | 0.18653 | 0.995 | 0.41374 |  |
|  | Diff-IVW | 0.932 | 0.13235 | 0.957 | 0.13592 | 0.97 | 0.05978 | 0.987 | 0.20213 |  |
|  | Diff-Egger | 0.919 | 0.44591 | 0.863 | 0.19995 | 0.726 | 0.40688 | 1 | 95.03062 |  |
|  | Diff-Median | 0.927 | 0.13779 | 0.992 | 0.14426 | 0.938 | 0.11250 | 0.972 | 0.42194 |  |
| Product | Prod-IVW_0_ | 0.229 | 0.12761 | 0.846 | 0.14355 | 0.535 | 0.06560 | 0.997 | 0.20510 |  |
|  | Prod-IVW | 0.932 | 0.13235 | 0.941 | 0.14031 | 0.993 | 0.04633 | 0.957 | 0.18184 |  |
|  | Prod-Egger | 0.919 | 0.44591 | 0.952 | 0.47006 | 0.893 | 0.14542 | 0.959 | 27.22627 |  |
|  | Prod-Median | 0.937 | 0.13889 | 0.959 | 0.14890 | 0.929 | 0.05351 | 0.957 | 0.24554 |  |
| **Table S19** Mean and mean squared error (MSE) of the estimate for Scenario 9$(\alpha=0.3, \beta=0.3, \delta=0.21,\tau=0.3, \rho=0.3)$ | | | | | | | | | |  |
| Method | | Total effect  $(\tau=0.3)$ | | Direct effect  $(\delta=0.21)$ | | Indirect effect  $(\alpha\beta=0.09)$ | | Mediation proportion  $(\rho=0.3)$ | |  |
|  |  | Mean | MSE | Mean | MSE | Mean | MSE | Mean | MSE |  |
| Difference | Diff-IVW_0_ | 0.51994 | 0.04881 | 0.34126 | 0.01800 | 0.17869 | 0.00823 | 0.34417 | 0.00347 |  |
|  | Diff-IVW | 0.50961 | 0.04450 | 0.34126 | 0.01800 | 0.16836 | 0.00635 | 0.33107 | 0.00203 |  |
|  | Diff-Egger | 0.83487 | 0.29310 | 0.42087 | 0.04633 | 0.41400 | 0.11064 | 0.49312 | 0.04026 |  |
|  | Diff-Median | 0.34042 | 0.00278 | 0.26542 | 0.00447 | 0.07500 | 0.00107 | 0.21983 | 0.01346 |  |
| Product | Prod-IVW_0_ | 0.51994 | 0.04881 | 0.28974 | 0.00700 | 0.23021 | 0.01985 | 0.44349 | 0.02166 |  |
|  | Prod-IVW | 0.50961 | 0.04450 | 0.30984 | 0.01067 | 0.19977 | 0.01220 | 0.39283 | 0.00952 |  |
|  | Prod-Egger | 0.83487 | 0.29310 | 0.39243 | 0.04134 | 0.44244 | 0.12765 | 0.53366 | 0.06069 |  |
|  | Prod-Median | 0.34042 | 0.00278 | 0.23064 | 0.00182 | 0.10978 | 0.00061 | 0.32585 | 0.00377 |  |
| **Table S20** Coverage rate (C.R.) and interval length (I.L.) of the 95% confidence interval for Scenario 9 | | | | | | | | | |  |
| Method | | Total effect  $(\tau=0.3)$ | | Direct effect  $(\delta=0.21)$ | | Indirect effect  $(\alpha\beta=0.09)$ | | Mediation proportion  $(\rho=0.3)$ | |  |
|  |  | C.R. | I.L. | C.R. | I.L. | C.R. | I.L. | C.R. | I.L. |  |
| Difference | Diff-IVW_0_ | 0 | 0.16443 | 0.058 | 0.17470 | 0.947 | 0.23997 | 1 | 0.39551 |  |
|  | Diff-IVW | 0 | 0.20264 | 0.058 | 0.17470 | 0.355 | 0.14599 | 0.999 | 0.24333 |  |
|  | Diff-Egger | 0 | 0.54661 | 0.027 | 0.24688 | 0.161 | 0.50369 | 0.39 | 0.35295 |  |
|  | Diff-Median | 0.917 | 0.15218 | 0.658 | 0.15840 | 0.991 | 0.14286 | 0.908 | 0.41151 |  |
| Product | Prod-IVW_0_ | 0 | 0.16443 | 0.776 | 0.19864 | 0 | 0.11136 | 0.27 | 0.25668 |  |
|  | Prod-IVW | 0 | 0.20264 | 0.723 | 0.23086 | 0 | 0.11037 | 0.968 | 0.26795 |  |
|  | Prod-Egger | 0 | 0.54661 | 0.957 | 0.68151 | 0 | 0.40560 | 0.922 | 0.60577 |  |
|  | Prod-Median | 0.852 | 0.15121 | 0.95 | 0.16557 | 0.859 | 0.06724 | 0.983 | 0.24850 |  |
| **Table S21** Mean and mean squared error (MSE) of the estimate for Scenario 10$(\alpha=0.3, \beta=0.3, \delta=0.21,\tau=0.3, \rho=0.3)$ | | | | | | | | | |  |
| Method | | Total effect  $(\tau=0.3)$ | | Direct effect  $(\delta=0.21)$ | | Indirect effect  $(\alpha\beta=0.09)$ | | Mediation proportion  $(\rho=0.3)$ | |  |
|  |  | Mean | MSE | Mean | MSE | Mean | MSE | Mean | MSE |  |
| Difference | Diff-IVW_0_ | 0.58452 | 0.08140 | 0.36027 | 0.02341 | 0.22425 | 0.01846 | 0.38406 | 0.00848 |  |
|  | Diff-IVW | 0.56204 | 0.06926 | 0.36027 | 0.02341 | 0.20177 | 0.01279 | 0.35957 | 0.00465 |  |
|  | Diff-Egger | 0.85694 | 0.31805 | 0.46041 | 0.06504 | 0.39653 | 0.10034 | 0.45958 | 0.02903 |  |
|  | Diff-Median | 0.35985 | 0.00489 | 0.28082 | 0.00655 | 0.07903 | 0.00129 | 0.21812 | 0.01508 |  |
| Product | Prod-IVW_0_ | 0.58452 | 0.08140 | 0.34031 | 0.01770 | 0.24421 | 0.02401 | 0.41840 | 0.01498 |  |
|  | Prod-IVW | 0.56204 | 0.06926 | 0.32052 | 0.01297 | 0.24152 | 0.02315 | 0.43048 | 0.01793 |  |
|  | Prod-Egger | 0.85694 | 0.31805 | 0.33893 | 0.02593 | 0.51800 | 0.18742 | 0.60919 | 0.10326 |  |
|  | Prod-Median | 0.35985 | 0.00489 | 0.23988 | 0.00252 | 0.11996 | 0.00116 | 0.33702 | 0.00483 |  |
| **Table S22** Coverage rate (C.R.) and interval length (I.L.) of the 95% confidence interval for Scenario 10 | | | | | | | | | |  |
| Method | | Total effect  $(\tau=0.3)$ | | Direct effect  $(\delta=0.21)$ | | Indirect effect  $(\alpha\beta=0.09)$ | | Mediation proportion  $(\rho=0.3)$ | |  |
|  |  | C.R. | I.L. | C.R. | I.L. | C.R. | I.L. | C.R. | I.L. |  |
| Difference | Diff-IVW_0_ | 0 | 0.18105 | 0.036 | 0.19966 | 0.492 | 0.26959 | 0.996 | 0.39179 |  |
|  | Diff-IVW | 0 | 0.22173 | 0.036 | 0.19966 | 0.043 | 0.16767 | 0.988 | 0.25872 |  |
|  | Diff-Egger | 0.002 | 0.60795 | 0.023 | 0.28624 | 0.396 | 0.56264 | 0.675 | 0.40597 |  |
|  | Diff-Median | 0.79 | 0.16428 | 0.514 | 0.17012 | 0.998 | 0.16474 | 0.944 | 0.44303 |  |
| Product | Prod-IVW_0_ | 0 | 0.18105 | 0.241 | 0.22152 | 0 | 0.12754 | 0.65 | 0.25435 |  |
|  | Prod-IVW | 0 | 0.22173 | 0.752 | 0.26027 | 0 | 0.13605 | 0.792 | 0.29654 |  |
|  | Prod-Egger | 0.002 | 0.60795 | 0.995 | 0.78289 | 0 | 0.49193 | 0.87 | 0.72852 |  |
|  | Prod-Median | 0.724 | 0.16165 | 0.926 | 0.17777 | 0.706 | 0.07373 | 0.981 | 0.25866 |  |
| **Table S23** Mean and mean squared error (MSE) of the estimate for Scenario 11$(\alpha=0.3, \beta=0.3, \delta=0.21,\tau=0.3, \rho=0.3)$ | | | | | | | | | |  |
| Method | | Total effect  $(\tau=0.3)$ | | Direct effect  $(\delta=0.21)$ | | Indirect effect  $(\alpha\beta=0.09)$ | | Mediation proportion  $(\rho=0.3)$ | |  |
|  |  | Mean | MSE | Mean | MSE | Mean | MSE | Mean | MSE |  |
| Difference | Diff-IVW_0_ | 0.58415 | 0.08121 | 0.36642 | 0.02526 | 0.21774 | 0.01676 | 0.37307 | 0.00670 |  |
|  | Diff-IVW | 0.56179 | 0.06915 | 0.36642 | 0.02526 | 0.19537 | 0.01143 | 0.34822 | 0.00343 |  |
|  | Diff-Egger | 0.85537 | 0.31584 | 0.45636 | 0.06310 | 0.39902 | 0.10161 | 0.46359 | 0.03032 |  |
|  | Diff-Median | 0.35936 | 0.00482 | 0.28667 | 0.00730 | 0.07269 | 0.00140 | 0.20045 | 0.01764 |  |
| Product | Prod-IVW_0_ | 0.58415 | 0.08121 | 0.34336 | 0.01850 | 0.24079 | 0.02299 | 0.41276 | 0.01367 |  |
|  | Prod-IVW | 0.56179 | 0.06915 | 0.32525 | 0.01400 | 0.23654 | 0.02170 | 0.42168 | 0.01571 |  |
|  | Prod-Egger | 0.85537 | 0.31584 | 0.39148 | 0.04170 | 0.46389 | 0.14426 | 0.54578 | 0.06743 |  |
|  | Prod-Median | 0.35936 | 0.00482 | 0.24644 | 0.00293 | 0.11293 | 0.00080 | 0.31758 | 0.00365 |  |
| **Table S24** Coverage rate (C.R.) and interval length (I.L.) of the 95% confidence interval for Scenario 11 | | | | | | | | | |  |
| Method | | Total effect  $(\tau=0.3)$ | | Direct effect  $(\delta=0.21)$ | | Indirect effect  $(\alpha\beta=0.09)$ | | Mediation proportion  $(\rho=0.3)$ | |  |
|  |  | C.R. | I.L. | C.R. | I.L. | C.R. | I.L. | C.R. | I.L. |  |
| Difference | Diff-IVW_0_ | 0 | 0.18080 | 0.017 | 0.19483 | 0.575 | 0.26587 | 0.999 | 0.38637 |  |
|  | Diff-IVW | 0 | 0.22149 | 0.017 | 0.19483 | 0.102 | 0.16797 | 0.997 | 0.25539 |  |
|  | Diff-Egger | 0.004 | 0.60668 | 0.021 | 0.28355 | 0.361 | 0.55939 | 0.648 | 0.40066 |  |
|  | Diff-Median | 0.766 | 0.16351 | 0.412 | 0.16711 | 0.996 | 0.16288 | 0.911 | 0.43765 |  |
| Product | Prod-IVW_0_ | 0 | 0.18080 | 0.197 | 0.22045 | 0 | 0.12604 | 0.697 | 0.25131 |  |
|  | Prod-IVW | 0 | 0.22149 | 0.71 | 0.26064 | 0 | 0.13717 | 0.894 | 0.29625 |  |
|  | Prod-Egger | 0.004 | 0.60668 | 0.985 | 0.78204 | 0.005 | 0.49224 | 0.971 | 0.70314 |  |
|  | Prod-Median | 0.722 | 0.16116 | 0.904 | 0.17677 | 0.824 | 0.07236 | 0.977 | 0.25036 |  |
| **Table S25** Mean and mean squared error (MSE) of the estimate for Scenario 1 for binary $Y (\alpha=0.3, \beta=0.3, \delta=0.21,\tau=0.3, \rho=0.3)$ | | | | | | | | | |  |
| Method | | Total effect  $(\tau=0.3)$ | | Direct effect  $(\delta=0.21)$ | | Indirect effect  $(\alpha\beta=0.09)$ | | Mediation proportion  $(\rho=0.3)$ | |  |
|  |  | Mean | MSE | Mean | MSE | Mean | MSE | Mean | MSE |  |
| Difference | Diff-IVW_0_ | 0.29571 | 0.00084 | 0.18091 | 0.00210 | 0.11480 | 0.00105 | 0.39201 | 0.01505 |  |
|  | Diff-IVW | 0.26635 | 0.00220 | 0.18091 | 0.00210 | 0.08545 | 0.00018 | 0.32604 | 0.00494 |  |
|  | Diff-Egger | 0.20701 | 0.02133 | 0.21113 | 0.00256 | -0.00412 | 0.01954 | -1.01878 | 863.73040 |  |
|  | Diff-Median | 0.25279 | 0.00391 | 0.18144 | 0.00280 | 0.07135 | 0.00093 | 0.28815 | 0.01137 |  |
| Product | Prod-IVW_0_ | 0.29571 | 0.00084 | 0.18910 | 0.00156 | 0.10661 | 0.00052 | 0.36440 | 0.00860 |  |
|  | Prod-IVW | 0.26635 | 0.00220 | 0.18565 | 0.00187 | 0.08071 | 0.00023 | 0.30828 | 0.00398 |  |
|  | Prod-Egger | 0.20701 | 0.02133 | 0.13530 | 0.01970 | 0.07171 | 0.00170 | 0.80579 | 188.11734 |  |
|  | Prod-Median | 0.25279 | 0.00391 | 0.17830 | 0.00297 | 0.07450 | 0.00044 | 0.30381 | 0.00673 |  |
| **Table S26** Coverage rate (C.R.) and interval length (I.L.) of the 95% confidence interval for Scenario 1 for binary $Y$ | | | | | | | | | |  |
| Method | | Total effect  $(\tau=0.3)$ | | Direct effect  $(\delta=0.21)$ | | Indirect effect  $(\alpha\beta=0.09)$ | | Mediation proportion  $(\rho=0.3)$ | |  |
|  |  | C.R. | I.L. | C.R. | I.L. | C.R. | I.L. | C.R. | I.L. |  |
| Difference | Diff-IVW_0_ | 0.953 | 0.11873 | 0.871 | 0.13936 | 1 | 0.18314 | 0.985 | 0.53564 |  |
|  | Diff-IVW | 0.842 | 0.13425 | 0.871 | 0.13936 | 0.943 | 0.05533 | 0.958 | 0.25727 |  |
|  | Diff-Egger | 0.88 | 0.45548 | 0.952 | 0.20514 | 0.853 | 0.41381 | 0.985 | 2870.3357 |  |
|  | Diff-Median | 0.817 | 0.18755 | 0.97 | 0.19449 | 0.984 | 0.13064 | 0.999 | 0.60577 |  |
| Product | Prod-IVW_0_ | 0.953 | 0.11873 | 0.913 | 0.13488 | 0.863 | 0.06385 | 0.912 | 0.26501 |  |
|  | Prod-IVW | 0.842 | 0.13425 | 0.9 | 0.14253 | 0.865 | 0.04767 | 0.947 | 0.24365 |  |
|  | Prod-Egger | 0.88 | 0.45548 | 0.912 | 0.48368 | 0.91 | 0.15964 | 0.956 | 1337.6426 |  |
|  | Prod-Median | 0.873 | 0.19068 | 0.933 | 0.20217 | 0.863 | 0.06695 | 0.957 | 0.36817 |  |
| **Table S27** Mean and mean squared error (MSE) of the estimate for Scenario 2 for binary $Y (\alpha=0.3, \beta=0.3, \delta=0.21,\tau=0.3, \rho=0.3)$ | | | | | | | | | |  |
| Method | | Total effect  $(\tau=0.3)$ | | Direct effect  $(\delta=0.21)$ | | Indirect effect  $(\alpha\beta=0.09)$ | | Mediation proportion  $(\rho=0.3)$ | |  |
|  |  | Mean | MSE | Mean | MSE | Mean | MSE | Mean | MSE |  |
| Difference | Diff-IVW_0_ | 0.29741 | 0.00090 | 0.18083 | 0.00212 | 0.11658 | 0.00111 | 0.39586 | 0.01537 |  |
|  | Diff-IVW | 0.26890 | 0.00215 | 0.18083 | 0.00212 | 0.08807 | 0.00021 | 0.33229 | 0.00533 |  |
|  | Diff-Egger | 0.24619 | 0.01523 | 0.20487 | 0.00288 | 0.04132 | 0.01221 | 0.03563 | 28.46871 |  |
|  | Diff-Median | 0.25611 | 0.00381 | 0.18026 | 0.00294 | 0.07586 | 0.00097 | 0.30041 | 0.01318 |  |
| Product | Prod-IVW_0_ | 0.29741 | 0.00090 | 0.18761 | 0.00165 | 0.10980 | 0.00063 | 0.37313 | 0.00965 |  |
|  | Prod-IVW | 0.26890 | 0.00215 | 0.18303 | 0.00208 | 0.08587 | 0.00017 | 0.32492 | 0.00477 |  |
|  | Prod-Egger | 0.24619 | 0.01523 | 0.12443 | 0.02162 | 0.12176 | 0.00392 | 0.71254 | 8.91463 |  |
|  | Prod-Median | 0.25611 | 0.00381 | 0.17598 | 0.00332 | 0.08013 | 0.00033 | 0.32324 | 0.00850 |  |
| **Table S28** Coverage rate (C.R.) and interval length (I.L.) of the 95% confidence interval for Scenario 2 for binary $Y$ | | | | | | | | | |  |
| Method | | Total effect  $(\tau=0.3)$ | | Direct effect  $(\delta=0.21)$ | | Indirect effect  $(\alpha\beta=0.09)$ | | Mediation proportion  $(\rho=0.3)$ | |  |
|  |  | C.R. | I.L. | C.R. | I.L. | C.R. | I.L. | C.R. | I.L. |  |
| Difference | Diff-IVW_0_ | 0.954 | 0.12118 | 0.858 | 0.13781 | 1 | 0.18358 | 0.982 | 0.52991 |  |
|  | Diff-IVW | 0.853 | 0.13649 | 0.858 | 0.13781 | 0.967 | 0.06456 | 0.965 | 0.26987 |  |
|  | Diff-Egger | 0.929 | 0.46262 | 0.958 | 0.21243 | 0.936 | 0.42030 | 0.976 | 92.80144 |  |
|  | Diff-Median | 0.842 | 0.18827 | 0.961 | 0.19174 | 0.995 | 0.14215 | 0.998 | 0.62803 |  |
| Product | Prod-IVW_0_ | 0.954 | 0.12118 | 0.904 | 0.13866 | 0.853 | 0.06725 | 0.916 | 0.27697 |  |
|  | Prod-IVW | 0.853 | 0.13649 | 0.891 | 0.14728 | 0.951 | 0.05509 | 0.98 | 0.27041 |  |
|  | Prod-Egger | 0.929 | 0.46262 | 0.923 | 0.51712 | 0.964 | 0.22801 | 0.993 | 39.35575 |  |
|  | Prod-Median | 0.874 | 0.19110 | 0.926 | 0.20353 | 0.921 | 0.06978 | 0.974 | 0.38463 |  |
| **Table S29** Mean and mean squared error (MSE) of the estimate for Scenario 3 for binary $Y (\alpha=0.3, \beta=0.3, \delta=0.21,\tau=0.3, \rho=0.3)$ | | | | | | | | | |  |
| Method | | Total effect  $(\tau=0.3)$ | | Direct effect  $(\delta=0.21)$ | | Indirect effect  $(\alpha\beta=0.09)$ | | Mediation proportion  $(\rho=0.3)$ | |  |
|  |  | Mean | MSE | Mean | MSE | Mean | MSE | Mean | MSE |  |
| Difference | Diff-IVW_0_ | 0.28358 | 0.00112 | 0.15768 | 0.00394 | 0.12590 | 0.00171 | 0.44836 | 0.02921 |  |
|  | Diff-IVW | 0.24714 | 0.00389 | 0.15768 | 0.00394 | 0.08946 | 0.00018 | 0.36819 | 0.00992 |  |
|  | Diff-Egger | 0.13385 | 0.04194 | 0.17790 | 0.00430 | -0.04405 | 0.03022 | -0.21908 | 114.84169 |  |
|  | Diff-Median | 0.22833 | 0.00708 | 0.15879 | 0.00472 | 0.06954 | 0.00114 | 0.31075 | 0.01665 |  |
| Product | Prod-IVW_0_ | 0.28358 | 0.00112 | 0.17071 | 0.00262 | 0.11287 | 0.00075 | 0.40231 | 0.01514 |  |
|  | Prod-IVW | 0.24714 | 0.00389 | 0.16179 | 0.00356 | 0.08535 | 0.00016 | 0.35178 | 0.00750 |  |
|  | Prod-Egger | 0.13385 | 0.04194 | 0.08526 | 0.03126 | 0.04858 | 0.00342 | 0.56419 | 68.29768 |  |
|  | Prod-Median | 0.22833 | 0.00708 | 0.15352 | 0.00540 | 0.07480 | 0.00046 | 0.34165 | 0.01258 |  |
| **Table S30** Coverage rate (C.R.) and interval length (I.L.) of the 95% confidence interval for Scenario 3 for binary $Y$ | | | | | | | | | |  |
| Method | | Total effect  $(\tau=0.3)$ | | Direct effect  $(\delta=0.21)$ | | Indirect effect  $(\alpha\beta=0.09)$ | | Mediation proportion  $(\rho=0.3)$ | |  |
|  |  | C.R. | I.L. | C.R. | I.L. | C.R. | I.L. | C.R. | I.L. |  |
| Difference | Diff-IVW_0_ | 0.97 | 0.14167 | 0.834 | 0.17154 | 1 | 0.22258 | 0.984 | 0.67202 |  |
|  | Diff-IVW | 0.807 | 0.16378 | 0.834 | 0.17154 | 0.99 | 0.07469 | 0.996 | 0.37608 |  |
|  | Diff-Egger | 0.815 | 0.55416 | 0.953 | 0.25319 | 0.854 | 0.50611 | 0.99 | 566.68034 |  |
|  | Diff-Median | 0.601 | 0.19668 | 0.924 | 0.20389 | 0.982 | 0.14097 | 0.999 | 0.77969 |  |
| Product | Prod-IVW_0_ | 0.97 | 0.14167 | 0.896 | 0.16146 | 0.878 | 0.07729 | 0.927 | 0.34443 |  |
|  | Prod-IVW | 0.807 | 0.16378 | 0.868 | 0.17341 | 0.961 | 0.05660 | 0.997 | 0.33756 |  |
|  | Prod-Egger | 0.815 | 0.55416 | 0.909 | 0.58619 | 0.843 | 0.18672 | 0.962 | 472.80218 |  |
|  | Prod-Median | 0.731 | 0.19887 | 0.849 | 0.21033 | 0.873 | 0.06823 | 0.982 | 0.45406 |  |
| **Table S31** Mean and mean squared error (MSE) of the estimate for Scenario 4 for binary $Y (\alpha=0.3, \beta=0.3, \delta=0.21,\tau=0.3, \rho=0.3)$ | | | | | | | | | |  |
| Method | | Total effect  $(\tau=0.3)$ | | Direct effect  $(\delta=0.21)$ | | Indirect effect  $(\alpha\beta=0.09)$ | | Mediation proportion  $(\rho=0.3)$ | |  |
|  |  | Mean | MSE | Mean | MSE | Mean | MSE | Mean | MSE |  |
| Difference | Diff-IVW_0_ | 0.31091 | 0.00102 | 0.20991 | 0.00120 | 0.10100 | 0.00056 | 0.32723 | 0.00592 |  |
|  | Diff-IVW | 0.28397 | 0.00149 | 0.20991 | 0.00120 | 0.07407 | 0.00044 | 0.26354 | 0.00415 |  |
|  | Diff-Egger | 0.28232 | 0.01744 | 0.24500 | 0.00464 | 0.03733 | 0.01439 | -0.01631 | 1.19313 |  |
|  | Diff-Median | 0.26391 | 0.00346 | 0.20086 | 0.00222 | 0.06306 | 0.00161 | 0.23981 | 0.01664 |  |
| Product | Prod-IVW_0_ | 0.31091 | 0.00102 | 0.19779 | 0.00121 | 0.11313 | 0.00072 | 0.36723 | 0.00780 |  |
|  | Prod-IVW | 0.28397 | 0.00149 | 0.21311 | 0.00137 | 0.07086 | 0.00047 | 0.25368 | 0.00471 |  |
|  | Prod-Egger | 0.28232 | 0.01744 | 0.23946 | 0.01578 | 0.04287 | 0.00305 | 0.17767 | 0.05586 |  |
|  | Prod-Median | 0.26391 | 0.00346 | 0.19762 | 0.00257 | 0.06629 | 0.00077 | 0.26037 | 0.00795 |  |
| **Table S32** Coverage rate (C.R.) and interval length (I.L.) of the 95% confidence interval for Scenario 4 for binary $Y$ | | | | | | | | | |  |
| Method | | Total effect  $(\tau=0.3)$ | | Direct effect  $(\delta=0.21)$ | | Indirect effect  $(\alpha\beta=0.09)$ | | Mediation proportion  $(\rho=0.3)$ | |  |
|  |  | C.R. | I.L. | C.R. | I.L. | C.R. | I.L. | C.R. | I.L. |  |
| Difference | Diff-IVW_0_ | 0.993 | 0.17566 | 0.988 | 0.16860 | 1 | 0.24356 | 1 | 0.66856 |  |
|  | Diff-IVW | 0.99 | 0.20238 | 0.988 | 0.16860 | 0.998 | 0.12552 | 0.993 | 0.37991 |  |
|  | Diff-Egger | 0.958 | 0.56334 | 0.949 | 0.25741 | 0.971 | 0.50359 | 0.982 | 5.95791 |  |
|  | Diff-Median | 0.919 | 0.20465 | 0.996 | 0.20048 | 0.954 | 0.15310 | 0.984 | 0.66310 |  |
| Product | Prod-IVW_0_ | 0.993 | 0.17566 | 0.993 | 0.19323 | 0.919 | 0.08034 | 0.995 | 0.33664 |  |
|  | Prod-IVW | 0.99 | 0.20238 | 0.995 | 0.20970 | 0.735 | 0.05454 | 0.899 | 0.27113 |  |
|  | Prod-Egger | 0.958 | 0.56334 | 0.972 | 0.57678 | 0.548 | 0.11779 | 0.681 | 1.10170 |  |
|  | Prod-Median | 0.916 | 0.20763 | 0.965 | 0.21726 | 0.688 | 0.06366 | 0.874 | 0.33569 |  |
| **Table S33** Mean and mean squared error (MSE) of the estimate for Scenario 5 for binary $Y (\alpha=0.3, \beta=0.3, \delta=0.21,\tau=0.3, \rho=0.3)$ | | | | | | | | | |  |
| Method | | Total effect  $(\tau=0.3)$ | | Direct effect  $(\delta=0.21)$ | | Indirect effect  $(\alpha\beta=0.09)$ | | Mediation proportion  $(\rho=0.3)$ | |  |
|  |  | Mean | MSE | Mean | MSE | Mean | MSE | Mean | MSE |  |
| Difference | Diff-IVW_0_ | 0.29507 | 0.00101 | 0.18406 | 0.00193 | 0.11100 | 0.00090 | 0.37956 | 0.01265 |  |
|  | Diff-IVW | 0.26037 | 0.00287 | 0.18406 | 0.00193 | 0.07631 | 0.00042 | 0.29695 | 0.00418 |  |
|  | Diff-Egger | 0.22714 | 0.02435 | 0.19332 | 0.00431 | 0.03382 | 0.01733 | -0.06214 | 43.52772 |  |
|  | Diff-Median | 0.23647 | 0.00650 | 0.17959 | 0.00321 | 0.05689 | 0.00241 | 0.23737 | 0.02884 |  |
| Product | Prod-IVW_0_ | 0.29507 | 0.00101 | 0.17510 | 0.00238 | 0.11997 | 0.00109 | 0.41135 | 0.01677 |  |
|  | Prod-IVW | 0.26037 | 0.00287 | 0.18182 | 0.00220 | 0.07854 | 0.00025 | 0.30772 | 0.00389 |  |
|  | Prod-Egger | 0.22714 | 0.02435 | 0.16830 | 0.01872 | 0.05883 | 0.00204 | 0.32026 | 7.83146 |  |
|  | Prod-Median | 0.23647 | 0.00650 | 0.16746 | 0.00446 | 0.06901 | 0.00064 | 0.30700 | 0.01043 |  |
| **Table S34** Coverage rate (C.R.) and interval length (I.L.) of the 95% confidence interval for Scenario 5 for binary $Y$ | | | | | | | | | |  |
| Method | | Total effect  $(\tau=0.3)$ | | Direct effect  $(\delta=0.21)$ | | Indirect effect  $(\alpha\beta=0.09)$ | | Mediation proportion  $(\rho=0.3)$ | |  |
|  |  | C.R. | I.L. | C.R. | I.L. | C.R. | I.L. | C.R. | I.L. |  |
| Difference | Diff-IVW_0_ | 0.991 | 0.18609 | 0.961 | 0.18624 | 1 | 0.26337 | 1 | 0.75114 |  |
|  | Diff-IVW | 0.97 | 0.21874 | 0.961 | 0.18624 | 0.997 | 0.13221 | 0.998 | 0.44411 |  |
|  | Diff-Egger | 0.952 | 0.60925 | 0.978 | 0.29773 | 0.964 | 0.53630 | 0.965 | 177.40000 |  |
|  | Diff-Median | 0.783 | 0.21438 | 0.963 | 0.20499 | 0.974 | 0.17381 | 0.998 | 0.91115 |  |
| Product | Prod-IVW_0_ | 0.991 | 0.18609 | 0.972 | 0.20596 | 0.883 | 0.08811 | 0.992 | 0.40348 |  |
|  | Prod-IVW | 0.97 | 0.21874 | 0.989 | 0.22923 | 0.956 | 0.06813 | 0.994 | 0.38074 |  |
|  | Prod-Egger | 0.952 | 0.60925 | 0.98 | 0.63063 | 0.81 | 0.15706 | 0.92 | 70.24357 |  |
|  | Prod-Median | 0.813 | 0.21629 | 0.921 | 0.22628 | 0.77 | 0.06616 | 0.954 | 0.43432 |  |
| **Table S35** Mean and mean squared error (MSE) of the estimate for Scenario 6 for binary $Y (\alpha=0.3, \beta=0.3, \delta=0.21,\tau=0.3, \rho=0.3)$ | | | | | | | | | |  |
| Method | | Total effect  $(\tau=0.3)$ | | Direct effect  $(\delta=0.21)$ | | Indirect effect  $(\alpha\beta=0.09)$ | | Mediation proportion  $(\rho=0.3)$ | |  |
|  |  | Mean | MSE | Mean | MSE | Mean | MSE | Mean | MSE |  |
| Difference | Diff-IVW_0_ | 0.29538 | 0.00102 | 0.18952 | 0.00164 | 0.10586 | 0.00072 | 0.36131 | 0.00991 |  |
|  | Diff-IVW | 0.26081 | 0.00284 | 0.18952 | 0.00164 | 0.07129 | 0.00058 | 0.27660 | 0.00443 |  |
|  | Diff-Egger | 0.22912 | 0.02338 | 0.19329 | 0.00434 | 0.03583 | 0.01648 | -0.08837 | 36.60038 |  |
|  | Diff-Median | 0.23744 | 0.00636 | 0.18488 | 0.00278 | 0.05256 | 0.00262 | 0.21732 | 0.02913 |  |
| Product | Prod-IVW_0_ | 0.29538 | 0.00102 | 0.17793 | 0.00217 | 0.11745 | 0.00094 | 0.40217 | 0.01456 |  |
|  | Prod-IVW | 0.26081 | 0.00284 | 0.18539 | 0.00200 | 0.07542 | 0.00033 | 0.29493 | 0.00369 |  |
|  | Prod-Egger | 0.22912 | 0.02338 | 0.18840 | 0.01680 | 0.04073 | 0.00332 | 0.39394 | 39.72826 |  |
|  | Prod-Median | 0.23744 | 0.00636 | 0.17407 | 0.00390 | 0.06337 | 0.00090 | 0.28064 | 0.00945 |  |
| **Table S36** Coverage rate (C.R.) and interval length (I.L.) of the 95% confidence interval for Scenario 6 for binary $Y$ | | | | | | | | | |  |
| Method | | Total effect  $(\tau=0.3)$ | | Direct effect  $(\delta=0.21)$ | | Indirect effect  $(\alpha\beta=0.09)$ | | Mediation proportion  $(\rho=0.3)$ | |  |
|  |  | C.R. | I.L. | C.R. | I.L. | C.R. | I.L. | C.R. | I.L. |  |
| Difference | Diff-IVW_0_ | 0.994 | 0.18619 | 0.974 | 0.18462 | 1 | 0.26230 | 1 | 0.75206 |  |
|  | Diff-IVW | 0.964 | 0.21876 | 0.974 | 0.18462 | 0.994 | 0.13224 | 0.997 | 0.44123 |  |
|  | Diff-Egger | 0.961 | 0.61042 | 0.967 | 0.29702 | 0.965 | 0.53648 | 0.963 | 556.09850 |  |
|  | Diff-Median | 0.811 | 0.21444 | 0.975 | 0.20422 | 0.964 | 0.17356 | 0.994 | 0.90736 |  |
| Product | Prod-IVW_0_ | 0.994 | 0.18619 | 0.984 | 0.20570 | 0.919 | 0.08727 | 0.997 | 0.39710 |  |
|  | Prod-IVW | 0.964 | 0.21876 | 0.99 | 0.22914 | 0.911 | 0.06779 | 0.987 | 0.37166 |  |
|  | Prod-Egger | 0.961 | 0.61042 | 0.982 | 0.62753 | 0.612 | 0.13880 | 0.802 | 765.01222 |  |
|  | Prod-Median | 0.814 | 0.21636 | 0.934 | 0.22535 | 0.603 | 0.06265 | 0.915 | 0.40272 |  |
| **Table S37** Mean and mean squared error (MSE) of the estimate for Scenario 7 for binary $Y (\alpha=0.3, \beta=0.3, \delta=0.21,\tau=0.3, \rho=0.3)$ | | | | | | | | | |  |
| Method | | Total effect  $(\tau=0.3)$ | | Direct effect  $(\delta=0.21)$ | | Indirect effect  $(\alpha\beta=0.09)$ | | Mediation proportion  $(\rho=0.3)$ | |  |
|  |  | Mean | MSE | Mean | MSE | Mean | MSE | Mean | MSE |  |
| Difference | Diff-IVW_0_ | 0.31181 | 0.00102 | 0.18527 | 0.00187 | 0.12654 | 0.00188 | 0.40873 | 0.01843 |  |
|  | Diff-IVW | 0.28406 | 0.00138 | 0.18527 | 0.00187 | 0.09879 | 0.00035 | 0.35189 | 0.00744 |  |
|  | Diff-Egger | 0.20929 | 0.02174 | 0.20822 | 0.00293 | 0.00107 | 0.01883 | -0.38028 | 120.72885 |  |
|  | Diff-Median | 0.26684 | 0.00289 | 0.18412 | 0.00266 | 0.08272 | 0.00094 | 0.31327 | 0.01311 |  |
| Product | Prod-IVW_0_ | 0.31181 | 0.00102 | 0.19053 | 0.00158 | 0.12128 | 0.00128 | 0.39267 | 0.01334 |  |
|  | Prod-IVW | 0.28406 | 0.00138 | 0.18102 | 0.00215 | 0.10304 | 0.00037 | 0.36791 | 0.00912 |  |
|  | Prod-Egger | 0.20929 | 0.02174 | 0.12871 | 0.02192 | 0.08058 | 0.00200 | 0.50092 | 21.06034 |  |
|  | Prod-Median | 0.26684 | 0.00289 | 0.18335 | 0.00274 | 0.08349 | 0.00028 | 0.32150 | 0.00732 |  |
| **Table S38** Coverage rate (C.R.) and interval length (I.L.) of the 95% confidence interval for Scenario 7 for binary $Y$ | | | | | | | | | |  |
| Method | | Total effect  $(\tau=0.3)$ | | Direct effect  $(\delta=0.21)$ | | Indirect effect  $(\alpha\beta=0.09)$ | | Mediation proportion  $(\rho=0.3)$ | |  |
|  |  | C.R. | I.L. | C.R. | I.L. | C.R. | I.L. | C.R. | I.L. |  |
| Difference | Diff-IVW_0_ | 0.937 | 0.11902 | 0.895 | 0.14093 | 0.996 | 0.18454 | 0.962 | 0.50998 |  |
|  | Diff-IVW | 0.931 | 0.13574 | 0.895 | 0.14093 | 0.938 | 0.07146 | 0.939 | 0.28163 |  |
|  | Diff-Egger | 0.871 | 0.46044 | 0.948 | 0.21410 | 0.859 | 0.41619 | 0.984 | 279.80178 |  |
|  | Diff-Median | 0.903 | 0.18849 | 0.974 | 0.19640 | 0.997 | 0.14825 | 0.999 | 0.62050 |  |
| Product | Prod-IVW_0_ | 0.937 | 0.11902 | 0.922 | 0.13927 | 0.611 | 0.07217 | 0.819 | 0.27980 |  |
|  | Prod-IVW | 0.931 | 0.13574 | 0.881 | 0.14894 | 0.913 | 0.06105 | 0.951 | 0.28414 |  |
|  | Prod-Egger | 0.871 | 0.46044 | 0.904 | 0.49762 | 0.935 | 0.18467 | 0.968 | 107.18395 |  |
|  | Prod-Median | 0.923 | 0.19157 | 0.949 | 0.20400 | 0.939 | 0.06984 | 0.98 | 0.36522 |  |
| **Table S39** Mean and mean squared error (MSE) of the estimate for Scenario 8 for binary $Y (\alpha=0.3, \beta=0.3, \delta=0.21,\tau=0.3, \rho=0.3)$ | | | | | | | | | |  |
| Method | | Total effect  $(\tau=0.3)$ | | Direct effect  $(\delta=0.21)$ | | Indirect effect  $(\alpha\beta=0.09)$ | | Mediation proportion  $(\rho=0.3)$ | |  |
|  |  | Mean | MSE | Mean | MSE | Mean | MSE | Mean | MSE |  |
| Difference | Diff-IVW_0_ | 0.34621 | 0.00299 | 0.20987 | 0.00129 | 0.13634 | 0.00259 | 0.39663 | 0.01427 |  |
|  | Diff-IVW | 0.30001 | 0.00118 | 0.20987 | 0.00129 | 0.09014 | 0.00017 | 0.30419 | 0.00312 |  |
|  | Diff-Egger | 0.18697 | 0.02606 | 0.23778 | 0.00392 | -0.05081 | 0.03081 | -1.19886 | 141.46719 |  |
|  | Diff-Median | 0.26139 | 0.00340 | 0.19554 | 0.00241 | 0.06585 | 0.00135 | 0.25585 | 0.01462 |  |
| Product | Prod-IVW_0_ | 0.34621 | 0.00299 | 0.23458 | 0.00168 | 0.11163 | 0.00070 | 0.32472 | 0.00334 |  |
|  | Prod-IVW | 0.30001 | 0.00118 | 0.21547 | 0.00133 | 0.08454 | 0.00017 | 0.28552 | 0.00293 |  |
|  | Prod-Egger | 0.18697 | 0.02606 | 0.12703 | 0.02217 | 0.05994 | 0.00260 | 0.46607 | 11.45584 |  |
|  | Prod-Median | 0.26139 | 0.00340 | 0.18651 | 0.00267 | 0.07488 | 0.00045 | 0.29516 | 0.00646 |  |
| **Table S40** Coverage rate (C.R.) and interval length (I.L.) of the 95% confidence interval for Scenario 8 for binary $Y$ | | | | | | | | | |  |
| Method | | Total effect  $(\tau=0.3)$ | | Direct effect  $(\delta=0.21)$ | | Indirect effect  $(\alpha\beta=0.09)$ | | Mediation proportion  $(\rho=0.3)$ | |  |
|  |  | C.R. | I.L. | C.R. | I.L. | C.R. | I.L. | C.R. | I.L. |  |
| Difference | Diff-IVW_0_ | 0.815 | 0.14602 | 0.969 | 0.16298 | 0.999 | 0.21893 | 0.994 | 0.53863 |  |
|  | Diff-IVW | 0.978 | 0.15717 | 0.969 | 0.16298 | 0.988 | 0.07201 | 0.978 | 0.27390 |  |
|  | Diff-Egger | 0.905 | 0.52986 | 0.946 | 0.24020 | 0.806 | 0.48378 | 1 | 379.54298 |  |
|  | Diff-Median | 0.92 | 0.19428 | 0.988 | 0.20139 | 0.968 | 0.13932 | 0.992 | 0.60560 |  |
| Product | Prod-IVW_0_ | 0.815 | 0.14602 | 0.96 | 0.16372 | 0.86 | 0.07386 | 0.983 | 0.25641 |  |
|  | Prod-IVW | 0.978 | 0.15717 | 0.968 | 0.16609 | 0.949 | 0.05332 | 0.944 | 0.23774 |  |
|  | Prod-Egger | 0.905 | 0.52986 | 0.94 | 0.55968 | 0.885 | 0.17625 | 0.937 | 90.97462 |  |
|  | Prod-Median | 0.911 | 0.19672 | 0.951 | 0.20819 | 0.868 | 0.06787 | 0.957 | 0.35960 |  |
| **Table S41** Mean and mean squared error (MSE) of the estimate for Scenario 9 for binary $Y (\alpha=0.3, \beta=0.3, \delta=0.21,\tau=0.3, \rho=0.3)$ | | | | | | | | | |  |
| Method | | Total effect  $(\tau=0.3)$ | | Direct effect  $(\delta=0.21)$ | | Indirect effect  $(\alpha\beta=0.09)$ | | Mediation proportion  $(\rho=0.3)$ | |  |
|  |  | Mean | MSE | Mean | MSE | Mean | MSE | Mean | MSE |  |
| Difference | Diff-IVW_0_ | 0.47283 | 0.03064 | 0.30981 | 0.01129 | 0.16301 | 0.00592 | 0.34592 | 0.00517 |  |
|  | Diff-IVW | 0.46356 | 0.02778 | 0.30981 | 0.01129 | 0.15375 | 0.00437 | 0.33327 | 0.00314 |  |
|  | Diff-Egger | 0.76088 | 0.22257 | 0.38113 | 0.03225 | 0.37975 | 0.09232 | 0.49431 | 0.04346 |  |
|  | Diff-Median | 0.35731 | 0.00574 | 0.26063 | 0.00505 | 0.09668 | 0.00121 | 0.27143 | 0.00954 |  |
| Product | Prod-IVW_0_ | 0.47283 | 0.03064 | 0.26383 | 0.00413 | 0.20899 | 0.01447 | 0.44388 | 0.02312 |  |
|  | Prod-IVW | 0.46356 | 0.02778 | 0.28191 | 0.00646 | 0.18165 | 0.00862 | 0.39384 | 0.01070 |  |
|  | Prod-Egger | 0.76088 | 0.22257 | 0.35915 | 0.03380 | 0.40172 | 0.10089 | 0.53566 | 0.06580 |  |
|  | Prod-Median | 0.35731 | 0.00574 | 0.23719 | 0.00367 | 0.12013 | 0.00126 | 0.34347 | 0.00766 |  |
| **Table S42** Coverage rate (C.R.) and interval length (I.L.) of the 95% confidence interval for Scenario 9 for binary $Y$ | | | | | | | | | |  |
| Method | | Total effect  $(\tau=0.3)$ | | Direct effect  $(\delta=0.21)$ | | Indirect effect  $(\alpha\beta=0.09)$ | | Mediation proportion  $(\rho=0.3)$ | |  |
|  |  | C.R. | I.L. | C.R. | I.L. | C.R. | I.L. | C.R. | I.L. |  |
| Difference | Diff-IVW_0_ | 0.001 | 0.16859 | 0.435 | 0.18800 | 0.98 | 0.25262 | 0.999 | 0.46271 |  |
|  | Diff-IVW | 0.034 | 0.20583 | 0.435 | 0.18800 | 0.657 | 0.13954 | 0.985 | 0.26944 |  |
|  | Diff-Egger | 0.036 | 0.56023 | 0.255 | 0.26676 | 0.363 | 0.51135 | 0.482 | 0.40394 |  |
|  | Diff-Median | 0.853 | 0.21363 | 0.872 | 0.21164 | 0.999 | 0.17922 | 0.999 | 0.46745 |  |
| Product | Prod-IVW_0_ | 0.001 | 0.16859 | 0.9 | 0.20200 | 0 | 0.11113 | 0.517 | 0.28517 |  |
|  | Prod-IVW | 0.034 | 0.20583 | 0.889 | 0.23200 | 0.001 | 0.10666 | 0.95 | 0.29138 |  |
|  | Prod-Egger | 0.036 | 0.56023 | 0.968 | 0.68215 | 0.004 | 0.38719 | 0.959 | 0.65996 |  |
|  | Prod-Median | 0.833 | 0.21173 | 0.936 | 0.22726 | 0.759 | 0.08226 | 0.985 | 0.31779 |  |
| **Table S43** Mean and mean squared error (MSE) of the estimate for Scenario 10 for binary $Y (\alpha=0.3, \beta=0.3, \delta=0.21,\tau=0.3, \rho=0.3)$ | | | | | | | | | |  |
| Method | | Total effect  $(\tau=0.3)$ | | Direct effect  $(\delta=0.21)$ | | Indirect effect  $(\alpha\beta=0.09)$ | | Mediation proportion  $(\rho=0.3)$ | |  |
|  |  | Mean | MSE | Mean | MSE | Mean | MSE | Mean | MSE |  |
| Difference | Diff-IVW_0_ | 0.52929 | 0.05333 | 0.32721 | 0.01511 | 0.20208 | 0.01322 | 0.38275 | 0.00956 |  |
|  | Diff-IVW | 0.50899 | 0.04477 | 0.32721 | 0.01511 | 0.18177 | 0.00884 | 0.35840 | 0.00536 |  |
|  | Diff-Egger | 0.77321 | 0.23433 | 0.41582 | 0.04565 | 0.35739 | 0.07955 | 0.45728 | 0.03054 |  |
|  | Diff-Median | 0.38407 | 0.00986 | 0.27411 | 0.00689 | 0.10995 | 0.00221 | 0.28546 | 0.01101 |  |
| Product | Prod-IVW_0_ | 0.52929 | 0.05333 | 0.30857 | 0.01091 | 0.22072 | 0.01743 | 0.41832 | 0.01583 |  |
|  | Prod-IVW | 0.50899 | 0.04477 | 0.29078 | 0.00784 | 0.21821 | 0.01670 | 0.43046 | 0.01875 |  |
|  | Prod-Egger | 0.77321 | 0.23433 | 0.30585 | 0.02256 | 0.46736 | 0.14682 | 0.61430 | 0.11178 |  |
|  | Prod-Median | 0.38407 | 0.00986 | 0.25366 | 0.00503 | 0.13041 | 0.00203 | 0.34612 | 0.00732 |  |
| **Table S44** Coverage rate (C.R.) and interval length (I.L.) of the 95% confidence interval for Scenario 10 for binary $Y$ | | | | | | | | | |  |
| Method | | Total effect  $(\tau=0.3)$ | | Direct effect  $(\delta=0.21)$ | | Indirect effect  $(\alpha\beta=0.09)$ | | Mediation proportion  $(\rho=0.3)$ | |  |
|  |  | C.R. | I.L. | C.R. | I.L. | C.R. | I.L. | C.R. | I.L. |  |
| Difference | Diff-IVW_0_ | 0 | 0.18176 | 0.356 | 0.20696 | 0.828 | 0.27554 | 1 | 0.44610 |  |
|  | Diff-IVW | 0.004 | 0.22124 | 0.356 | 0.20696 | 0.298 | 0.16182 | 0.985 | 0.28601 |  |
|  | Diff-Egger | 0.057 | 0.60942 | 0.178 | 0.29836 | 0.558 | 0.56021 | 0.72 | 0.45906 |  |
|  | Diff-Median | 0.667 | 0.22254 | 0.818 | 0.21914 | 0.999 | 0.20245 | 0.997 | 0.48439 |  |
| Product | Prod-IVW_0_ | 0 | 0.18176 | 0.641 | 0.22115 | 0 | 0.12584 | 0.742 | 0.27907 |  |
|  | Prod-IVW | 0.004 | 0.22124 | 0.895 | 0.25654 | 0 | 0.12946 | 0.85 | 0.31787 |  |
|  | Prod-Egger | 0.057 | 0.60942 | 0.992 | 0.76667 | 0.002 | 0.46321 | 0.923 | 0.79128 |  |
|  | Prod-Median | 0.687 | 0.21947 | 0.913 | 0.23652 | 0.599 | 0.08786 | 0.987 | 0.31207 |  |
| **Table S45** Mean and mean squared error (MSE) of the estimate for Scenario 11 for binary $Y (\alpha=0.3, \beta=0.3, \delta=0.21,\tau=0.3, \rho=0.3)$ | | | | | | | | | |  |
| Method | | Total effect  $(\tau=0.3)$ | | Direct effect  $(\delta=0.21)$ | | Indirect effect  $(\alpha\beta=0.09)$ | | Mediation proportion  $(\rho=0.3)$ | |  |
|  |  | Mean | MSE | Mean | MSE | Mean | MSE | Mean | MSE |  |
| Difference | Diff-IVW_0_ | 0.52893 | 0.05312 | 0.33194 | 0.01612 | 0.19699 | 0.01212 | 0.37314 | 0.00796 |  |
|  | Diff-IVW | 0.50859 | 0.04454 | 0.33194 | 0.01612 | 0.17665 | 0.00796 | 0.34830 | 0.00425 |  |
|  | Diff-Egger | 0.77417 | 0.23434 | 0.40988 | 0.04307 | 0.36429 | 0.08306 | 0.46601 | 0.03317 |  |
|  | Diff-Median | 0.38335 | 0.00948 | 0.27947 | 0.00741 | 0.10387 | 0.00196 | 0.26970 | 0.01151 |  |
| Product | Prod-IVW_0_ | 0.52893 | 0.05312 | 0.31117 | 0.01134 | 0.21776 | 0.01667 | 0.41283 | 0.01447 |  |
|  | Prod-IVW | 0.50859 | 0.04454 | 0.29486 | 0.00842 | 0.21373 | 0.01560 | 0.42174 | 0.01651 |  |
|  | Prod-Egger | 0.77417 | 0.23434 | 0.35334 | 0.03218 | 0.42083 | 0.11419 | 0.55042 | 0.07357 |  |
|  | Prod-Median | 0.38335 | 0.00948 | 0.26066 | 0.00537 | 0.12269 | 0.00147 | 0.32546 | 0.00534 |  |
| **Table S46** Coverage rate (C.R.) and interval length (I.L.) of the 95% confidence interval for Scenario 11 for binary $Y$ | | | | | | | | | |  |
| Method | | Total effect  $(\tau=0.3)$ | | Direct effect  $(\delta=0.21)$ | | Indirect effect  $(\alpha\beta=0.09)$ | | Mediation proportion  $(\rho=0.3)$ | |  |
|  |  | C.R. | I.L. | C.R. | I.L. | C.R. | I.L. | C.R. | I.L. |  |
| Difference | Diff-IVW_0_ | 0 | 0.18184 | 0.283 | 0.20278 | 0.867 | 0.27247 | 0.999 | 0.44100 |  |
|  | Diff-IVW | 0.003 | 0.22093 | 0.283 | 0.20278 | 0.4 | 0.16165 | 0.986 | 0.28169 |  |
|  | Diff-Egger | 0.046 | 0.60915 | 0.185 | 0.29664 | 0.505 | 0.55739 | 0.678 | 0.44952 |  |
|  | Diff-Median | 0.665 | 0.22228 | 0.769 | 0.21650 | 1 | 0.20142 | 0.999 | 0.48064 |  |
| Product | Prod-IVW_0_ | 0 | 0.18184 | 0.604 | 0.22032 | 0 | 0.12426 | 0.787 | 0.27567 |  |
|  | Prod-IVW | 0.003 | 0.22093 | 0.886 | 0.25660 | 0 | 0.13011 | 0.895 | 0.31662 |  |
|  | Prod-Egger | 0.046 | 0.60915 | 0.981 | 0.76512 | 0.023 | 0.46094 | 0.987 | 0.75377 |  |
|  | Prod-Median | 0.688 | 0.21919 | 0.909 | 0.23545 | 0.734 | 0.08563 | 0.987 | 0.29913 |  |

# References

1. Burgess S, Bowden J. Integrating summarized data from multiple genetic variants in Mendelian randomization: bias and coverage properties of inverse-variance weighted methods. *arXiv preprint arXiv:151204486.* 2015.

2. Burgess S, Thompson DJ, Rees JM, Day FR, Perry JR, Ong KK. Dissecting causal pathways using Mendelian randomization with summarized genetic data: application to age at menarche and risk of breast cancer. *Genetics.* 2017;207(2):481-487.

3. Oehlert GW. A note on the delta method. *The American Statistician.* 1992;46(1):27-29.

4. Bowden J, Del Greco M F, Minelli C, Davey Smith G, Sheehan NA, Thompson JR. Assessing the suitability of summary data for two-sample Mendelian randomization analyses using MR-Egger regression: the role of the I2 statistic. *International journal of epidemiology.* 2016;45(6):1961-1974.

5. Burgess S, Butterworth A, Thompson SG. Mendelian randomization analysis with multiple genetic variants using summarized data. *Genet Epidemiol.* 2013;37(7):658-665.

6. VanderWeele TJ, Vansteelandt S. Odds ratios for mediation analysis for a dichotomous outcome. *American journal of epidemiology.* 2010;172(12):1339-1348.

7. Burgess S. Estimating and contextualizing the attenuation of odds ratios due to non collapsibility. *Communications in Statistics-Theory and Methods.* 2017;46(2):786-804.

8. Carter AR, Sanderson E, Hammerton G, et al. Mendelian randomisation for mediation analysis: current methods and challenges for implementation. *European journal of epidemiology.* 2021;36(5):465-478.

9. Pulit SL, Stoneman C, Morris AP, et al. Meta-analysis of genome-wide association studies for body fat distribution in 694 649 individuals of European ancestry. *Hum Mol Genet.* 2019;28(1):166-174.

10. Nikpay M, Goel A, Won HH, et al. A comprehensive 1,000 Genomes-based genome-wide association meta-analysis of coronary artery disease. *Nat Genet.* 2015;47(10):1121-1130.

11. Mahajan A, Taliun D, Thurner M, et al. Fine-mapping type 2 diabetes loci to single-variant resolution using high-density imputation and islet-specific epigenome maps. *Nat Genet.* 2018;50(11):1505-1513.

12. Wootton RE, Richmond RC, Stuijfzand BG, et al. Evidence for causal effects of lifetime smoking on risk for depression and schizophrenia: a Mendelian randomisation study. *Psychol Med.* 2020;50(14):2435-2443.

13. Hemani G, Zheng J, Elsworth B, et al. The MR-Base platform supports systematic causal inference across the human phenome. *elife.* 2018;7:e34408.

14. Scott RA, Scott LJ, Mägi R, et al. An expanded genome-wide association study of type 2 diabetes in Europeans. *Diabetes.* 2017;66(11):2888-2902.

15. Sanderson E, Spiller W, Bowden J. Testing and correcting for weak and pleiotropic instruments in two-sample multivariable Mendelian randomization. *Stat Med.* 2021;40(25):5434-5452.
